# Hematopoietic differentiation persists in human iPSCs defective in de novo DNA methylation

**DOI:** 10.1186/s12915-022-01343-x

**Published:** 2022-06-15

**Authors:** Olivia Cypris, Julia Franzen, Joana Frobel, Philipp Glück, Chao-Chung Kuo, Stephani Schmitz, Selina Nüchtern, Martin Zenke, Wolfgang Wagner

**Affiliations:** 1 Helmholtz-Institute for Biomedical Engineering, RWTH Aachen University Medical School, Pauwelsstraße 20, 52074 Aachen, North-Rhine Westphalia Germany; 2 Institute for Stem Cell Biology, RWTH Aachen University Medical School, 52074 Aachen, North-Rhine Westphalia Germany; 3 Center for Integrated Oncology Aachen Bonn Cologne Düsseldorf (CIO ABCD), 52074 Aachen, North-Rhine Westphalia Germany

**Keywords:** DNMT3A, Epigenetics, DNA methylation, CRISPR/Cas9, Induced pluripotent stem cells, Mesenchymal differentiation, Hematopoietic differentiation

## Abstract

**Background:**

DNA methylation is involved in the epigenetic regulation of gene expression during developmental processes and is primarily established by the DNA methyltransferase 3A (DNMT3A) and 3B (DNMT3B). *DNMT3A* is one of the most frequently mutated genes in clonal hematopoiesis and leukemia, indicating that it plays a crucial role for hematopoietic differentiation. However, the functional relevance of Dnmt3a for hematopoietic differentiation and hematological malignancies has mostly been analyzed in mice, with the specific role for human hematopoiesis remaining elusive. In this study, we therefore investigated if DNMT3A is essential for hematopoietic differentiation of human induced pluripotent stem cells (iPSCs).

**Results:**

We generated iPSC lines with knockout of either exon 2, 19, or 23 and analyzed the impact of different *DNMT3A* exon knockouts on directed differentiation toward mesenchymal and hematopoietic lineages. Exon 19^−/−^ and 23^−/−^ lines displayed an almost entire absence of de novo DNA methylation during mesenchymal and hematopoietic differentiation. Yet, differentiation efficiency was only slightly reduced in exon 19^−/−^ and rather increased in exon 23^−/−^ lines, while there was no significant impact on gene expression in hematopoietic progenitors (iHPCs). Notably, *DNMT3A*^−/−^ iHPCs recapitulate some DNA methylation patterns of acute myeloid leukemia (AML) with *DNMT3A* mutations. Furthermore, multicolor genetic barcoding revealed growth advantage of exon 23^−/−^ iHPCs in a syngeneic competitive differentiation assay.

**Conclusions:**

Our results demonstrate that iPSCs with homozygous knockout of different exons of *DNMT3A* remain capable of mesenchymal and hematopoietic differentiation—and exon 23^−/−^ iHPCs even gained growth advantage—despite loss of almost the entire de novo DNA methylation. Partial recapitulation of DNA methylation patterns of AML with *DNMT3A* mutations by our *DNMT3A* knockout iHPCs indicates that our model system can help to elucidate mechanisms of clonal hematopoiesis.

**Supplementary Information:**

The online version contains supplementary material available at 10.1186/s12915-022-01343-x.

## Background

DNA methylation (DNAm) is tightly regulated during cellular differentiation [1]. De novo methylation is primarily established by two DNA methyltransferases: DNMT3A and DNMT3B [2]. The latter is rather active in early development and specifically methylates minor satellite repeats, while DNMT3A was suggested to be more important in later development [2]. DNMT3A has five different protein-coding splice variants which might have different functional roles and are spliced in tissue- and disease-specific manner [3, 4]. So far, it is largely unclear how DNAm is targeted to specific sites in the genome.

DNMT3A seems to be of particular relevance for hematopoietic differentiation, since it frequently reveals heterozygous mutations in acute myeloid leukemia (AML) and other hematological malignancies [5, 6]. In AML, about 65% of the *DNMT3A* mutations are located in the hotspot R882 in exon 23 [7, 8]. This mutation is believed to reduce enzyme activity by blocking its ability to form active tetramers with the wildtype form [9] and to induce aberrant DNAm patterns due to conformational changes in the enzyme [8]. Furthermore, *DNMT3A* holds the most frequent driver mutations in clonal hematopoiesis of indeterminate potential (CHIP) [10].

The functional relevance of Dnmt3a was investigated in homozygous knockout mice already more than 20 years ago, demonstrating that development remained possible despite undersize at birth and death at about 4 weeks of age [2]. More recent analysis of murine *Dnmt3a* knockout models revealed an increased self-renewal capacity of hematopoietic stem cells (HSCs) with reduced differentiation capacity [11]. Heterozygous knockouts had only a subtle effect on methylation in mice with a bias for myeloid differentiation and higher propensity for malignant transformation [12]. Other studies reported that knocking out *Dnmt3a* in HSCs and transplanting them into mice lead to development of a wide range of myeloid and lymphoid malignancies [13–15].

In contrast, relatively few studies addressed the consequences of *DNMT3A* knockout in human cells. In human embryonic stem cells, no immediate negative effects of DNMT3A^−/−^ could be observed on downstream differentiation, although global loss of DNA methylation was observed after many passages with focal areas of hypermethylation [16]. Recently, it has been demonstrated that human-induced pluripotent stem cells (iPSCs) with knockout of *DNMT3A* can be differentiated into cardiomyocytes with similar efficiency as wildtype controls [17]. However, these iPSC-derived cardiomyocytes showed global hypomethylation with differences in contractile behavior and an aberrant activation of glucose and lipid metabolism [17].

In this study, we investigated the functional relevance of DNMT3A for hematopoietic differentiation of human iPSCs. To this end, we generated iPSC lines with knockout of exon 2, which contains a start codon for transcripts 1, 3, and 4. Alternatively, we removed exon 19, which has also been targeted in the abovementioned studies, or exon 23 that comprises the frequently mutated hotspot R882. We then analyzed the effect on growth and differentiation of iPSCs toward mesenchymal and hematopoietic lineages. We found only a moderate impact on differentiation with no significant changes in gene expression despite severe impact on de novo DNA methylation. Additionally, exon 23 knockout clones gained growth advantage over wildtype cells. Thus, de novo DNA methylation during early differentiation seems to be dependent on DNMT3A and disruption of the gene may be involved in competitive clonal advantages.

## Results

### Knockout of *DNMT3A* exons does not evoke phenotypic changes in human iPSCs

**Fig. 1:**
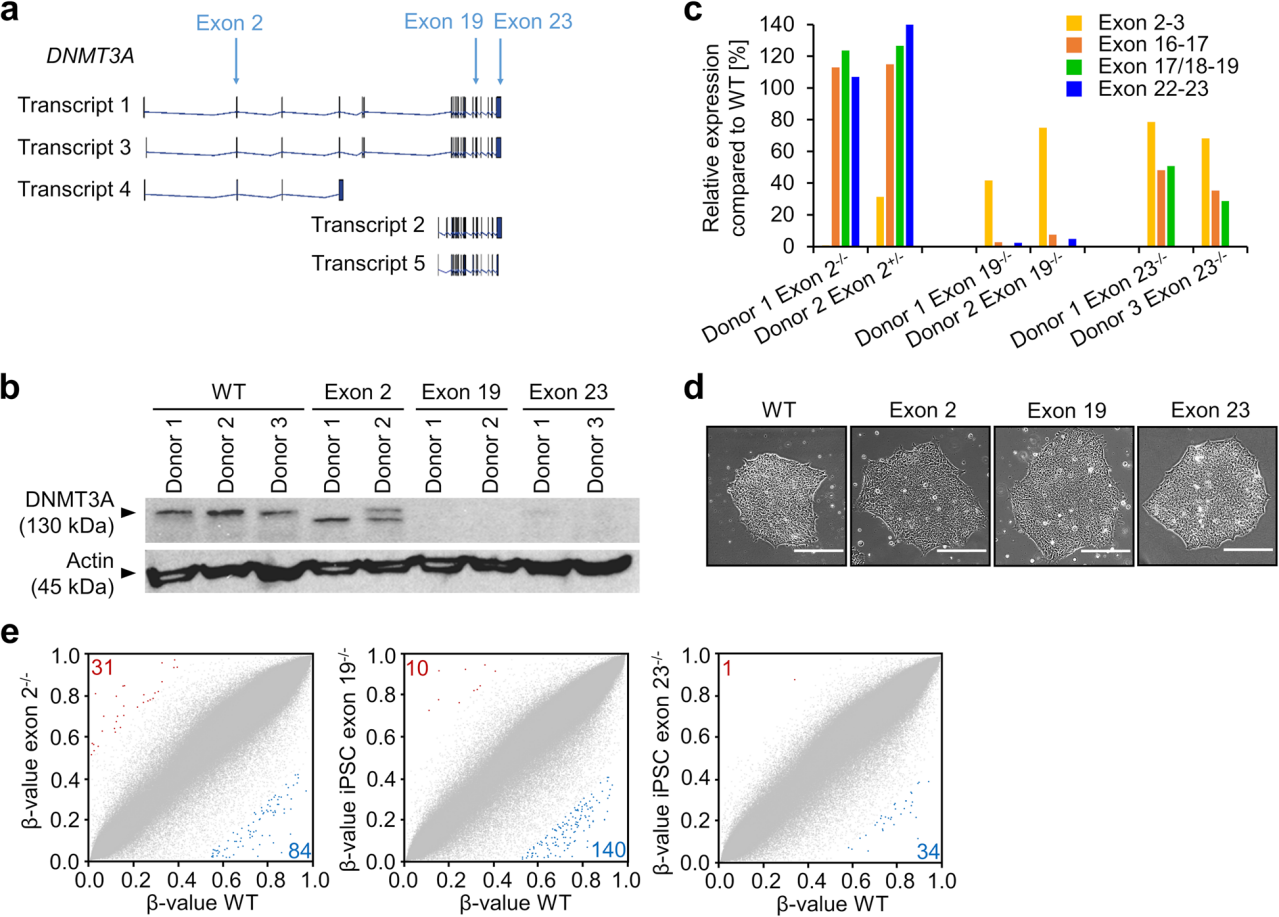
Characterization of different *DNMT3A* exon knockouts in iPSCs. **a** Schematic overview of the protein coding *DNMT3A* splice variants. Arrows indicate CRISPR/Cas9n target sites. **b** Western blot analysis of DNMT3A upon knockout of exon 2, exon 19, or exon 23. Arrow corresponds to height of transcript 1/3. WT = wildtype. **c** Semiquantitative RT-PCR analysis of expression of *DNMT3A* exons (normalized to *GAPDH*, measured in duplicate). **d** Representative phase contrast images of iPSC colonies with *DNMT3A* knockouts and WT. Scale bar = 150 μm. **e** Scatter plots show DNAm levels (*β*-values) for all measured CpG sites (grey) when comparing WT *versus* knockout of exons 2, 19, or 23, respectively. For exon 2, we only present results for the homozygous knockout of exon 2, whereas for exons 19 and 23 the mean of two homozygous knockout lines is presented. CpGs with more than 50% hypermethylation (red) or hypomethylation (blue) are indicated

To generate knockouts of different *DNMT3A* exons, we used iPSC lines from three donors for CRIPSR/Cas9n targeting of the intron/exon boundaries of exon 2 and exon 19, or excision of exon 23 (Fig. 1a; Additional file 1: Fig. S1). For exon 2, we achieved a homozygous truncation of *DNMT3A* for donor 1, due to a new in frame start codon in exon 3, and a heterozygous knockout for donor 2. Furthermore, we generated two homozygous knockout lines for exon 19 and two homozygous knockout lines for exon 23, as determined by Western blot analysis (Fig. 1b; Additional file 1: Fig. S2). These results were further substantiated by quantitative RT-PCR (Fig. 1c).

*DNMT3A* knockouts did not reveal negative effects on growth and differentiation of iPSCs. All clones showed typical iPSC-like morphology (Fig. 1d), expressed the pluripotency markers OCT3/4 and TRA1-60, and were able to differentiate toward endodermal, mesodermal, and ectodermal lineages (Additional file 1: Fig. S3a, b). To assess the impact of DNMT3A loss on DNA methylation, we used the Infinium Methylation EPIC BeadChip technology. Multidimensional scaling (MDS) indicated that DNAm profiles were similar in wildtype and *DNMT3A* knockout iPSC lines (Additional file 1: Fig. S3c). Pairwise comparison showed that only few CpGs had a clear conversion of DNAm levels (at least 50% gain or loss in DNAm) upon knockout, while there was no systematic off-set in DNAm levels (Fig. 1e, Additional file 2: Tab. S1). Taken together, we were able to successfully knock out three different exons of *DNMT3A* in human iPSCs and this did not evoke pronounced effects on DNAm levels while in a pluripotent cell state.

### *DNMT3A* knockouts impair de novo DNAm in iPSC-derived mesenchymal cells

**Fig. 2:**
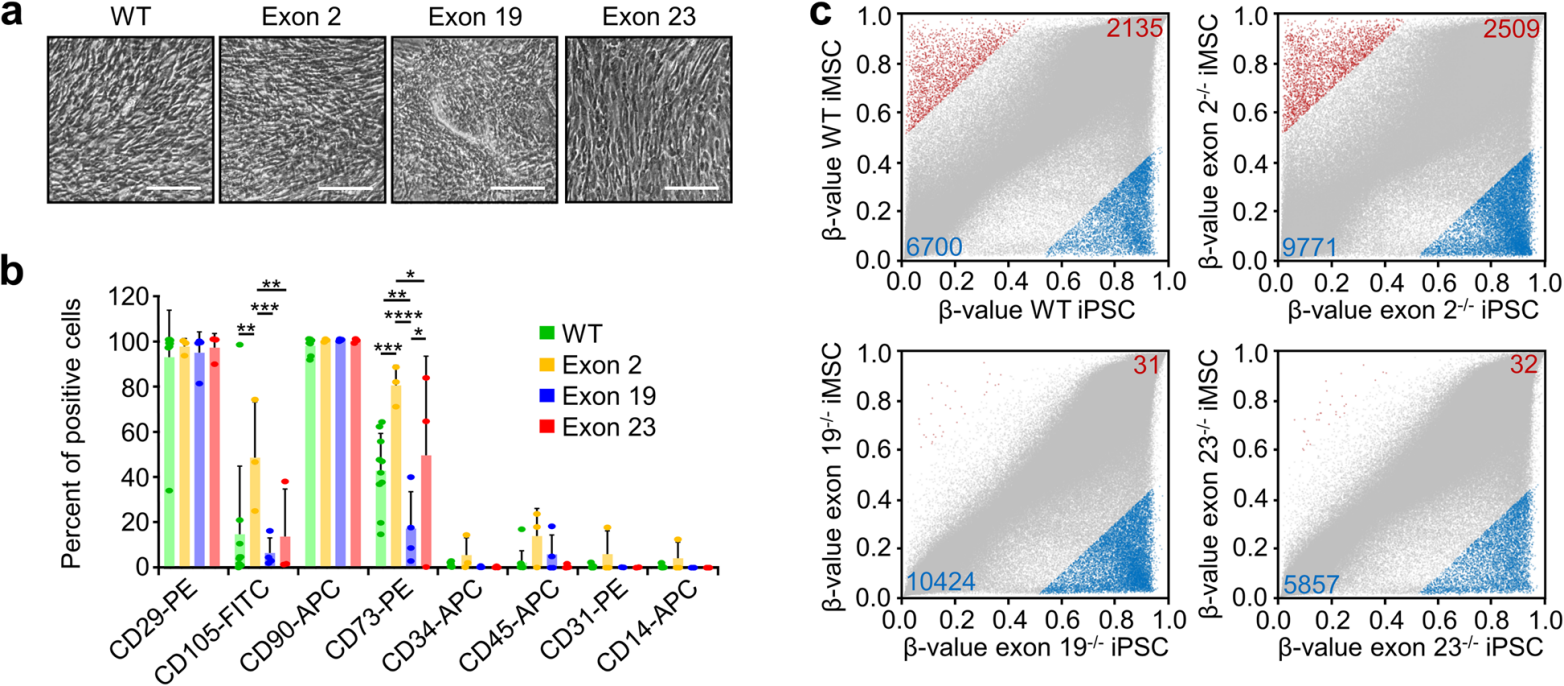
Characterization of iPSC-derived mesenchymal stromal cells. **a** Representative phase contrast images of iPSC-derived mesenchymal stromal cells (iMSCs) at day 35 for wildtype (WT) and knockout lines. Scale bar = 250 μm. **b** Flow cytometric analysis of surface markers of iMSCs at day 35. Significance was estimated by 2-way ANOVA with Tukey’s post hoc test; *n* = 10 for WT, *n* = 3 for exon 2 (exon 2^−/−^ and exon 2^+/−^), *n* = 4 for exon 19, and *n* = 3 for exon 23 (*P < 0.05; **P < 0.01, ***P < 0.001, ****P < 0.0001; mean ± SD). **c** Scatter plots of DNAm levels (*β*-values) for all measured CpG sites (grey) in iMSCs derived from homozygous knockout lines for exon 2 (*n* = 1), exon 19 (*n* = 2), or exon 23 (*n* = 2), in comparison to corresponding WT lines. CpGs with more than 50% hyper- (red) and hypomethylation (blue) are indicated. Darker blue and red spots represent promoter-associated CpGs

To test the impact of *DNMT3A* knockouts on directed differentiation, we initially differentiated all iPSC lines toward mesenchymal stromal cells (iMSCs) that constitute an important part of the hematopoietic niche. After 5 weeks, differentiated cells of all iPSC lines revealed typical fibroblastoid morphology (Fig. 2a) and many cells upregulated immunophenotypic markers for MSCs, albeit less pronounced for CD105 and CD73 in exon 19^−/−^ clones (Fig. 2b, Additional file 1: Fig. S4a). The differentiation of wildtype iPSCs toward iMSCs was associated with distinct DNAm changes of more than 50% at many CpGs: wildtype iMSCs gained DNAm at 2135 CpGs and lost methylation at 6700 CpGs (Fig. 2c; Additional file 3: Tab. S2). Notably, hypermethylated CpGs were enriched in promoter regions of genes in functional categories associated with mesenchymal differentiation, such as skeletal system development, osteoblast development, and cartilage development, indicating that de novo DNAm during iMSC differentiation is indeed lineage specific (Additional file 1: Fig. S4b). A similar number of differentially methylated CpGs were observed for the exon 2^−/−^ clone (2509 hyper- and 9771 hypomethylated CpGs) and most of them were overlapping with the DNAm changes of wildtype clones (Additional file 1: Fig. S4c). This might be anticipated, due to a new start codon in exon 3 maintaining the functional MTase-domain. In contrast, exon 19^−/−^ and exon 23^−/−^ clones hardly gained DNAm during differentiation toward iMSCs (only 31 and 32 CpGs, respectively), while hypomethylation followed a similar pattern as in wildtype iPSCs (10424 and 5857 CpGs, respectively). In fact, there was a very high overlap of hypomethylated CpGs in wildtype, exon 2^−/−^, exon 19^−/−^, and exon 23^−/−^ cells (Additional file 1: Fig. S4c). Our results demonstrate that despite similarities in the molecular phenotype of iMSCs there was hardly any de novo DNAm during differentiation of iPSCs with knockout of *DNMT3A* exon 19 and exon 23 toward the mesenchymal lineage.

### Hematopoietic differentiation is in tendency decreased in exon 19^−/−^ clones and increased in exon 23^−/−^ clones

**Fig. 3:**
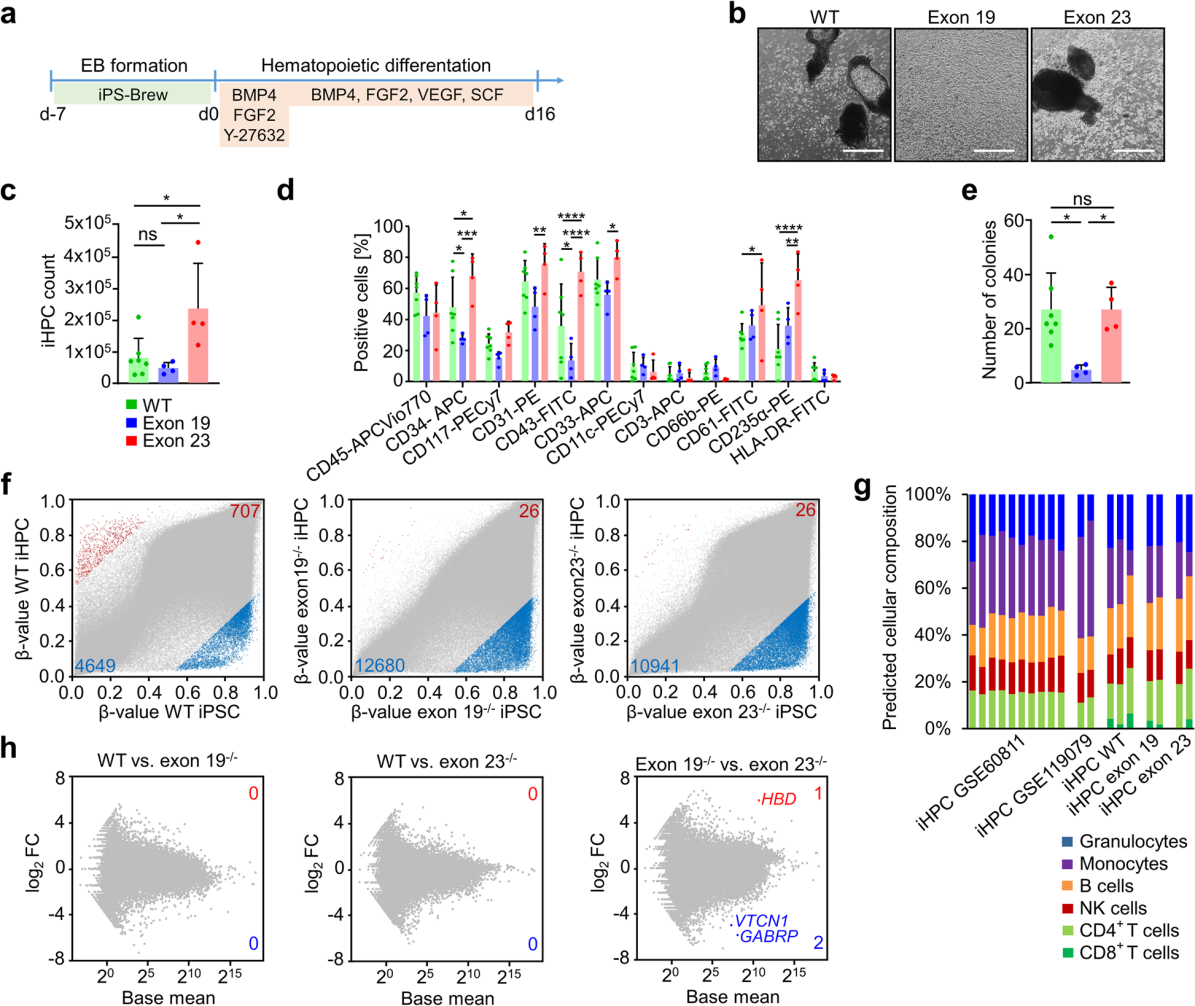
iPSC-derived hematopoietic progenitor cells. **a** Schematic representation of the protocol used for hematopoietic differentiation of iPSCs. **b** Representative phase contrast images of produced hematopoietic progenitor cells on day 16 of hematopoietic differentiation. Scale bar = 500 μm. **c** Absolute count of harvested iHPCs per well. Statistics were calculated with 1-way ANOVA and Tukey’s post hoc test; *n* = 7 for wildtype (WT), *n* = 4 for each knockout (*P < 0.05; mean ± SD). **d** Surface marker expression of hematopoietic progenitors. Statistics were calculated with 2-way ANOVA and Tukey’s post hoc test; *n* = 7 for WT, *n* = 4 for each knockout (*P < 0.05; **P < 0.01, ***P < 0.001, ****P < 0.0001; mean ± SD). **e** Total number of colonies formed in CFU assays. Statistics were calculated with 1-way ANOVA and Tukey’s post hoc test; *n* = 7 for WT, *n* = 4 for each knockout (*P < 0.05; mean ± SD). **f** Scatter plots show *β*-values for all measured CpG sites (grey) when comparing the mean of wildtype iHPCs, exon 19^−/−^ iHPCs or exon 23^−/−^ iHPCs with their respective iPSC counterparts. Hypermethylated CpGs are depicted in red, hypomethylated CpGs in blue with delta mean *β*-value >0.5 or <−0.5. CpG sites associated with promotor regions are shown without transparency. **g** A deconvolution algorithm [18] was used to estimate the composition of different mature hematopoietic cell types based on the DNAm profiles of our iHPCs, those of Nishizawa et al. [19] and of our previously published iHPC data [20]. **h** Gene expression changes compared between WT iHPCs and exon 19^−/−^ or exon 23^−/−^ iHPCs depicted as log_2_ fold change against the mean of normalized counts of all samples regarding sequencing depth (=base mean). Significantly differentially expressed genes are highlighted

Mutations in *DNMT3A* are frequently observed in hematopoietic malignancies and therefore we investigated if hematopoietic differentiation was affected in iPSCs with *DNMT3A* knockouts in exon 19 or 23 (Fig. 3a). We did not further consider the exon 2^−/−^ clone, since we did not have a homozygous biological replicate and no effects on DNAm were observed in iMSCs. After 16 days, all clones produced non-adherent cells with typical hematological morphology (Fig. 3b; Additional file 1: Fig. S5a) with a higher frequency in exon 23^−/−^ clones (Fig. 3c). Furthermore, in all clones, many of the differentiated cells expressed hematopoietic surface markers, albeit slightly less cells were positive for CD31, CD33, CD34, and CD43 in exon 19^−/−^ lines (Fig. 3d; Additional file 1: Fig. S5b), whereas CD61 and CD235α expressing cells were rather increased in exon 23^−/−^ lines. A direct comparison of exon 19^−/−^ and exon 23^−/−^ iHPCs revealed significantly higher proportions of CD31, CD33, CD34, CD43, and CD235a positive cells in the latter. We then tested the colony forming unit (CFU) potential in methylcellulose. In exon 19^−/−^ clones, the CFU frequency was significantly reduced in comparison to wildtype and exon 23^−/−^ clones and biased for CFU macrophage colonies (Fig. 3e; Additional file 1: Fig. S5c, d). To estimate if proliferation might be decreased in the exon 19^−/−^ clones that produced in tendency less hematopoietic cells, we analyzed expression of cell cycle-related genes and observed decreased expression of G2M and S phase genes in exon 19^−/−^ iHPCs, although differences in gene expression were not significant (Additional file 1: Fig. S5e). Overall, all iPSC lines were capable of differentiating toward hematopoietic lineage, but the propensity was reduced in exon 19^−/−^ as compared to wildtype or exon 23^−/−^ clones.

### Impaired de novo DNAm during hematopoietic differentiation is not reflected by the transcriptome

We subsequently analyzed DNAm changes after 16 days of hematopoietic differentiation. After differentiation, wildtype clones revealed strong DNAm changes (more than 50%) at many CpGs: 707 CpGs were hypermethylated and 4649 CpGs hypomethylated. In contrast, only 26 CpGs became hypermethylated in *DNMT3A* exon 19^−/−^, while 12680 CpGs became hypomethylated with the same thresholds. A similar effect was observed for *DNMT3A* exon 23^−/−^ lines (26 CpGs hyper- and 10941 CpGs hypomethylated; Fig. 3f; Additional file 4: Tab. S3). The overlap of CpGs with pronounced gains and losses in DNAm was very high between wildtype and knockout lines (Additional file 1: Fig. S6a). Furthermore, wildtype *versus* knockout iHPC clones revealed much higher DNAm at many CpGs in wildtype, whereas this was hardly observed in exon 19^−/−^ or exon 23^−/−^ (Additional file 1: Fig. S6b; Additional file 5: Tab. S4). Thus, *DNMT3A* knockout in either exon 19 or exon 23 clearly impairs de novo DNAm during hematopoietic differentiation of iPSCs.

The differences in hematopoietic differentiation between exon 19^−/−^ and exon 23^−/−^ clones might be attributed to different DNA methylation profiles. Overall, only 110 CpGs were more than 50% hypomethylated and 353 CpGs were more than 50% hypermethylated in exon 23^−/−^ *versus* exon 19^−/−^ lines. Very similar results were observed when we only compared the two samples of donor 1, to prevent impact of different genetic backgrounds (Additional file 1: Fig. S7a, b).

To estimate if *DNMT3A* knockouts affected differentiation toward specific hematopoietic lineages, we used a deconvolution algorithm to determine the composition of hematopoietic subsets based on DNAm profiles [18] (Fig. 3g). DNAm profiles of wildtype iHPCs indicated multilineage differentiation, similar to previously published iHPC profiles [19, 20]. Despite the impact of exon 19^−/−^ and exon 23^−/−^ on de novo DNAm, estimates for cellular composition were similar between wildtype and knockout iHPCs. This might be attributed to the finding that cell-type specific DNAm patterns of such signatures are rather hypomethylated [18, 21], and the hypomethylation was largely maintained in *DNMT3A* knockouts. Alternatively, we focused on individual CpGs that are specifically hypomethylated in granulocytes, monocytes, B cells, CD4, CD8, or NK cells [20, 22]. Overall, DNAm patterns at these lineage-specific sites were similar in wildtype and *DNMT3A* knockout iHPCs (Additional file 1: Fig. S6c).

To investigate if impaired de novo DNAm in *DNMT3A* knockouts was also reflected on transcriptomic level, we performed RNA-sequencing analysis. Unexpectedly, there were no significant differences in wildtype *versus* either exon 19^−/−^ or exon 23^−/−^ clones (Wald test; *P* < 0.05; Fig. 3h; Additional file 6: Tab. S5). Furthermore, even those genes that showed a difference of more than 50% on DNAm levels did not reveal a clear tendency for differential expression, even if we focused on DNAm changes in the promoter regions (Additional file 1: Fig. S6d). When we compared differential gene expression of iHPCs between exon 19^−/−^ or exon 23^−/−^ clones, only three genes reached significance (Hemoglobin Subunit Delta (*HBD*), V-Set Domain Containing T Cell Activation Inhibitor 1 (*VTCN1*), and Gamma-Aminobutyric Acid Type A Receptor Subunit Pi (*GABRP*; Fig. 3h). Taken together, loss of de novo methylation in exon 19^−/−^ and exon 23^−/−^ clones was not clearly reflected in differential gene expression.

### Impact of *DNMT3A* knockouts on specific DNAm changes

**Fig. 4:**
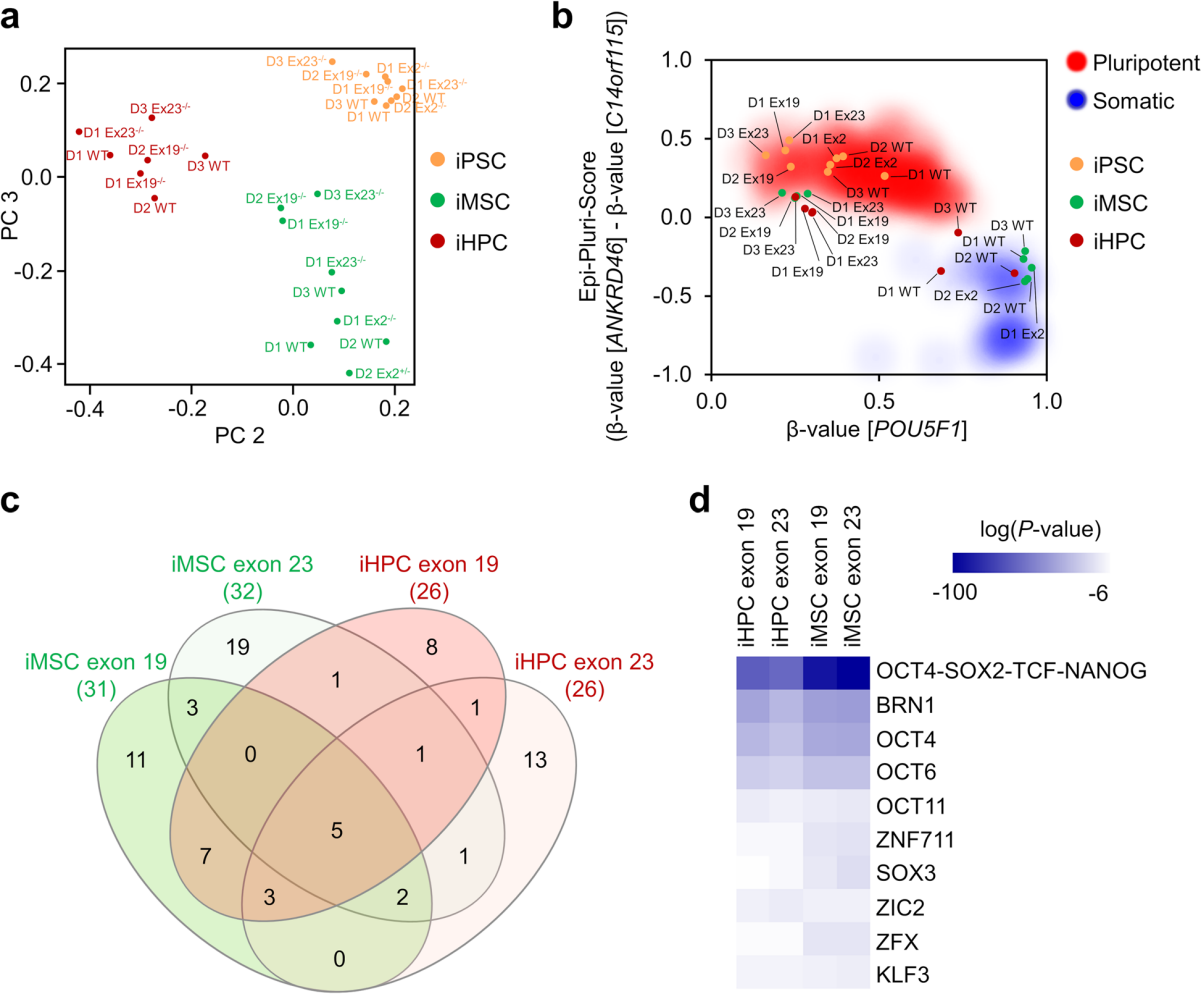
Comparison of DNA methylation profiles of all iPSCs, iMSCs and iHPCs. **a** Principal component analysis of global DNA methylation profiles of all iPSC lines and derived iMSCs or iHPCs (D = donor, WT = wildtype, Ex = exon). **b** Epi-Pluri-Score analysis of all generated cell lines and their progeny. **c** Venn diagram showing overlap of CpGs that were >50% hypermethylated during differentiation of iPSCs into iMSCs or iHPCs (mean of two biological replica, each). **d** Heatmap showing the top 10 transcription factor motifs enriched in these hypermethylated CpGs (compared to corresponding iPSCs)

To gain better insight into how global DNAm patterns change in iMSCs and iHPCs, we performed a principal component analysis (PCA). Despite the marked block of de novo DNAm, there are still cell-type specific changes, which can be particularly attributed to focal hypomethylation (Fig. 4a). Notably, many pluripotency-associated DNAm patterns, which are usually lost during differentiation, were maintained in iMSCs and iHPCs of exon 19^−/−^ and exon 23^−/−^ lines. This is exemplified by the Epi-Pluri-Score analysis, which resembles a targeted epigenetic signature to discern pluripotent and non-pluripotent cells [23], and methylation in the *POU5F1* promoter (Fig. 4b; Additional file 1: Fig. S8a, b). We have previously described that age-associated DNAm patterns are reversed during reprogramming into iPSCs [24] and all of our iPSC, iMSC, and iHPC lines maintained this rejuvenated epigenetic phenotype (Additional file 1: Fig. S8c).

Recently, it has been reported that genomic regions with low DNA methylation, termed long DNA methylation canyons, can form large loops connecting anchor loci of inter-chromosomal interaction [25]. *Dnmt3a* knockout mice demonstrated erosion of methylation canyons near developmental regulatory genes, like *HOX* clusters, or in association with HSC self-renewal genes [11]. Such analysis of large hypomethylated regions is better suited for whole genome bisulfite sequencing data, but we could also detect these canyons in our EPIC BeadChip data. In analogy to the previous studies in mice, particularly the canyon borders became hypomethylated in exon 19^−/−^ and exon 23^−/−^ knockout lines, as exemplarily depicted for the canyons in *PAX6* and *GATA2* (Additional file 1: Fig. S8d).

Finally, we focused on those few CpGs that still got highly methylated even without DNMT3A activity. These CpGs revealed a highly significant overlap for iMSCs and iHPCs (hypergeometric distribution; exon 19^−/−:^ *P* < 10^−60^, exon 23^−/−^: *P* < 10^−33^), and for exon 19^−/−^ and exon 23^−/−^ (iMSCs: *P* < 10^−36^, iHPCs: *P* < 10^−38^) (Fig. 4c). Most of these CpGs were located in enhancer regions. When we performed transcription factor binding motif analysis within a 100 bp window around the hypermethylated CpG sites, there was significant enrichment of pluripotency and development-associated transcription factors, which may indicate a pluripotency-associated regulatory mechanism possibly involved in the residual de novo DNAm during differentiation toward iMSCs or iHPCs (Fig. 4d).

### DNAm associated with *DNMT3A* mutations in AML is recapitulated in knockout iHPCs

**Fig. 5:**
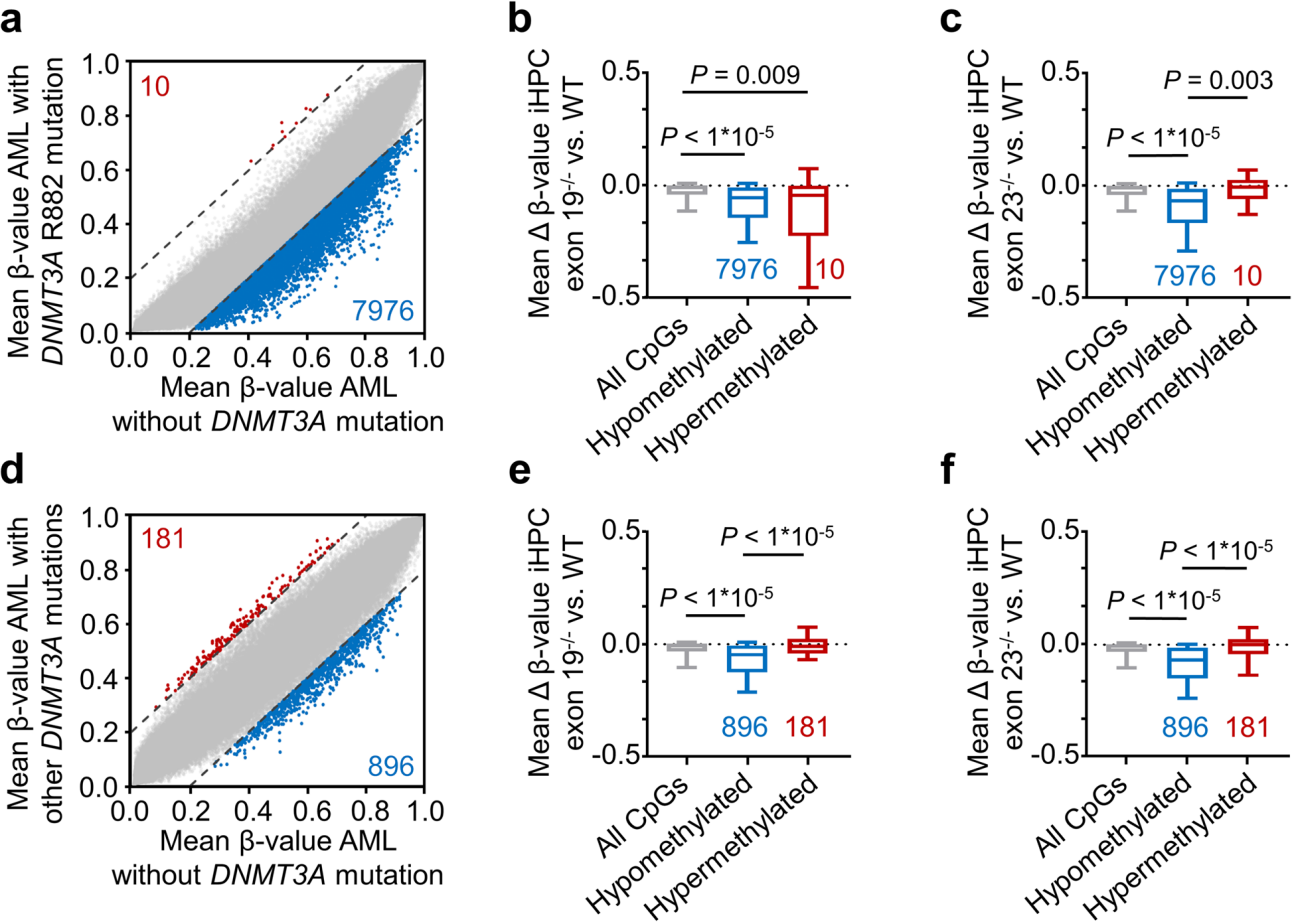
DNA methylation associated with *DNMT3A* mutations in acute myeloid leukemia. **a** Scatter plot of DNAm levels in acute myeloid leukemia (AML) with *DNMT3A* R882 mutation (*n* = 18) plotted against data of patients without *DNMT3A* mutations (*n* = 101). The numbers of CpGs beyond the 20% threshold of differential DNAm are depicted. **b** Box-whiskers plot (upper and lower quartiles, center line: median, whiskers: 10–90 percentile) showing mean hypo- and hypermethylation of the selected CpGs in (**a**) in the comparison *DNMT3A* exon 19^−/−^ *versus* wildtype (WT) iHPCs (mean of two biological replica). **c** Box-whiskers plot of mean hypo- and hypermethylation of the selected CpGs in (**a**) in the comparison of *DNMT3A* exon 23^−/−^ *versus* WT. **d** Scatter plot of DNAm levels in AML with *DNMT3A* mutations other than R882 (*n* = 15) plotted against data of patients without *DNMT3A* mutations. **e**, **f** Box-whiskers plots of differentially methylated CpGs in AML with non-R882 mutations in *DNMT3A* in analogy to (**b**, **c**). All statistics were performed with 1-way ANOVA and Tukey’s post hoc test

To better understand if DNAm changes of our *DNMT3A* knockout iPSC clones are related to aberrant DNAm patterns in AML patients with *DNMT3A* mutations, we utilized 134 DNAm datasets of AML patients from The Cancer Genome Atlas (TCGA) without mutation in *DNMT3A* (*n* = 101), with R882 mutations (*n* = 18), or with other *DNMT3A* mutations (*n* = 15). AML patients with the R882 mutations revealed global hypomethylation, as compared to AML patients without *DNMT3A* mutation (Fig. 5a): 7976 CpGs were more than 20% hypomethylated in R882 patients, whereas only 10 CpGs were 20% higher methylated. Notably, these hypomethylated CpGs showed on average also lower DNAm in our exon 19^−/−^ or exon 23^−/−^ iHPCs *versus* wildtype iHPCs (*P* < 10^−5^ as compared to all other CpGs for both comparisons; Fig. 5b, c). When we compared DNAm patterns in AML patients with other *DNMT3A* mutations to AML patients without *DNMT3A* mutations, the general hypomethylation was much less pronounced and only 896 CpGs revealed 20% lower DNAm with *DNMT3A* mutation, while 181 CpGs had at least 20% higher DNAm levels (Fig. 5d). Again, these hypomethylated CpGs were also hypomethylated in in our exon 19^−/−^ or exon 23^−/−^ iHPCs *versus* wildtype iHPCs (*P* < 10^−5^ for both comparisons, Fig. 5e, f). Thus, the DNAm patterns in AML patients that are related to *DNMT3A* mutations (R882 as well as other mutations) are partly recapitulated by our iPSC knockout models.

### Exon 23 knockout shows growth advantage during competitive hematopoietic differentiation

**Fig. 6:**
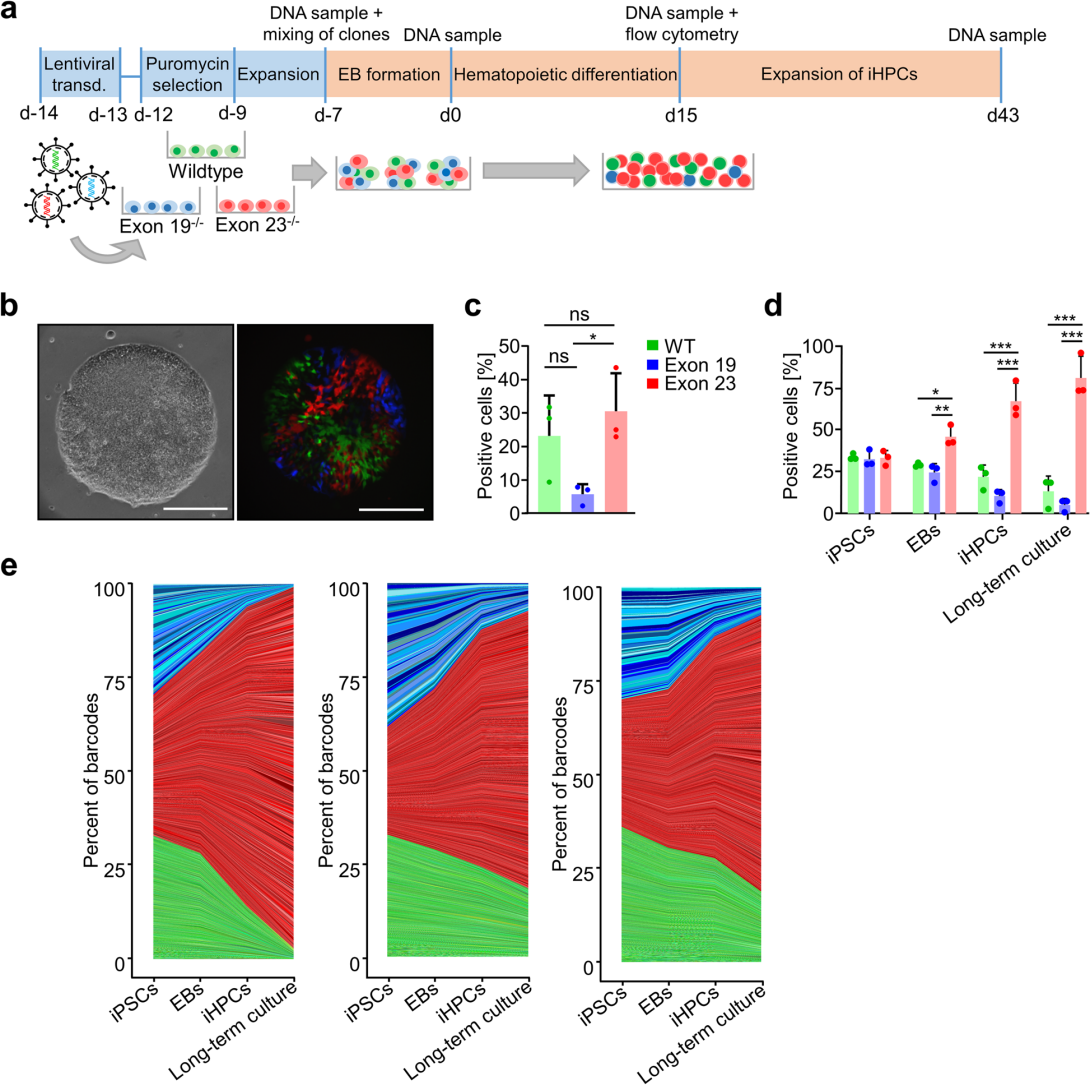
Competitive growth advantage of exon 23^−/−^ cells during hematopoietic differentiation. **a** Syngeneic wildtype, exon 19^−/−^ and exon 23^−/−^ iPSCs were transduced with a lentiviral barcoding system and the fluorophores Venus, Cerulean, and mCherry, respectively. The schematic representation depicts competitive growth in co-culture during embryoid body (EB) formation, hematopoietic differentiation, and additional long-term culture (*n* = 3, all donor 1). **b** Representative phase contrast and fluorescence microscopic images of EBs with mixed populations at day −4 (day 3 of EB growth). **c** Flow cytometry analysis of iHPCs after 15 days of hematopoietic differentiation (only cells with a clear fluorescent signal were considered). *n* = 3 (mean ± SD). **d** Alternatively, we analyzed the fluorophore-specific genetic barcodes by amplicon sequencing after mixing of the iPSCs (d-7), in EBs (d0), in iHPCs (d15), and after additional long-term expansion (d43). *n* = 3 (mean ± SD). **e** Area plots show clonal growth during differentiation and expansion of the three replicates. The graded colors depict corresponding unique molecular identifiers of the lentiviral barcoding. All statistics were performed with 1-way ANOVA and Tukey’s post hoc test. *P < 0.05, **P < 0.01, ***P < 0.001

We next wanted to better understand subclonal development during hematopoietic differentiation of iPSCs with and without *DNMT3A* knockouts. To this end, we transduced wildtype, exon 19^−/−^, or exon 23^−/−^ clones with lentiviral vectors containing unique molecular identifiers and the fluorophores Venus, Cerulean, or mCherry (Fig. 6a). The three syngeneic iPSC lines were then mixed with equal cell numbers and seeded on the microcontact-printed plates for EB formation (Fig. 6b). After hematopoietic differentiation, flow cytometric analysis demonstrated growth disadvantage of Cerulean labeled exon 19^−/−^ clones (Fig. 6c). Furthermore, we analyzed the fluorophore-specific genetic barcodes by amplicon deep-sequencing and exon 23^−/−^ cells had clear growth advantage with increasing fractions upon EB formation, iHPC production, and after additional long-term culture expansion for 28 days, whereas counts for exon 19^−/−^ cells decreased with time (Fig. 6d). These findings are in line with the higher iHPC numbers in both exon 23 knockouts, as indicated in Fig. 3c. However, exon 19^−/−^ clones did not reveal significant decline as compared to wildtype cells, even after 50 days in culture. Subsequently, the fractions of corresponding unique molecular identifier were tracked over the different time points demonstrating that hematopoietic differentiation of iPSCs is a multiclonal event (Fig. 6e). While some exon 23^−/−^ barcodes made up to 1.51% of all reads, there was no evidence for a clear oligo clonal composition after long-term expansion.

## Discussion

So far, the sequel of losing DNMT3A activity for hematopoietic development has mostly been investigated in the murine system by knocking out exon 19 or exon 18–20 of *Dnmt3a* with the Cre/loxP system [26, 27], or by targeting exon 19 with CRISPR/Cas9 [28]. Two groups established *DNMT3A* knockouts in human ESCs and iPSCs by targeting exon 19 [16, 17], but they did not describe their hematopoietic differentiation potential. iPSCs are a valuable model system for hematopoietic differentiation, because they facilitate systematic and clonal analysis in homogenous cell preparations [29].

Our results demonstrate that iPSCs with knockout of *DNMT3A* remain capable of differentiating toward hematopoietic lineages. We did not directly determine proliferation rates, but there may be evidence that exon 19^−/−^ lines have in tendency a slightly lower proliferation than wildtype clones, as suggested by expression of cell cycle-related genes and multicolor barcode analysis. Despite the pronounced differences in DNAm profiles of wildtype and *DNMT3A*^−/−^ clones, we did not find any significant differences in gene expression, indicating that de novo DNA methylation is not essential for differentiation into iHPCs. However, it is possible that deeper sequencing or more replicates would provide a more detailed view on the gene expression changes. It also needs to be considered that DNAm patterns of wildtype and knockout clone-derived iPSCs, iHPCs, and iMSCs still clustered together, probably due to cell-type specific hypomethylation, which may be functionally more relevant. Furthermore, other epigenetic modifications, such as histone modifications and chromatin conformation, may be primary drivers of directed cellular differentiation.

There are many different site-specific *DNMT3A* mutations in leukemia and CHIP [5, 7], and therefore, it might be anticipated that targeting of specific exons is of functional relevance. Exon 2 mutations in AML have been described before but its functional impact remains unclear because this exon is not associated with a functional protein domain [30, 31]. Mutations in exon 19 are highly abundant and may result in a functional loss [32, 33]. Exon 19 encodes parts of the methyltransferase domain and can form hydrogen bonds with DNA for its binding and stabilization during methylation activity [34]. More research has been performed for mutations in exon 23, particularly the hotspot mutation R882, which often results in loss of DNAm [8, 32]. This region is also involved in the methyltransferase domain and it is essential for interacting with the DNA backbone during DNMT3A-DNMT3A tetramer formation [34]. Our results support the notion that there may be functional differences between the different *DNMT3A* knockouts, but it needs to be considered that for each exon only two knockout lines have been generated and there is notorious variation in the differentiation potential of such cell lines. Furthermore, there were hardly differences in the DNA methylation profiles of iHPCs with exon 19^−/−^ and exon 23^−/−^. Additional research is therefore necessary to ultimately answer the question how aberrant splicing and specific mutations affect hematopoietic differentiation.

Only very few CpGs consistently gained DNAm in our exon 19^−/−^ and exon 23^−/−^ clones. They revealed a highly significant overlap in iMSCs and iHPCs and were associated with pluripotency- and development-associated transcription factor binding sites. Whether a pluripotency-associated regulatory mechanism might be involved in the residual de novo DNA methylation during differentiation needs to be further tested. In primary hematopoiesis, Dnmt3a has been shown to be a critical participant in the epigenetic silencing of HSC regulatory genes, thereby enabling efficient differentiation [26]. In contrast, Dnmt3b is also highly abundant in HSCs, but rather in a catalytically inactivated form with just some residual activity [2, 35]. The de novo methylation of these few CpGs might therefore be attributed to DNMT3B.

Acute myeloid leukemia is associated with characteristic aberrations in the DNA methylation pattern [36]. Patient samples with the hotspot mutation R882 revealed prominent hypomethylation compared to other *DNMT3A* mutations, as described before [9]. Our results demonstrate that iPSC-derived model iHPCs can recapitulate some epigenetic effects of R882-mutated and even in non-R882-mutated AML. Thus, aspects of AML-related DNAm patterns can be modeled by our system, which may be useful for further mechanistic analysis.

Lastly, we investigated if our iPSC model system also reveals subclonal dominance of *DNMT3A* knockouts in a competitive differentiation assay with long-term culture. In fact, exon 23^−/−^ iHPCs revealed growth advantage over wildtype and exon 19^−/−^ lines but there was no evidence for dominant subclones. In contrast, the lentiviral barcoding system showed dominant subclones in iMSCs, even without modifications in *DNMT3A* [37]. It is conceivable that differentiation of iHPCs is more homogeneous than iMSCs or that the effects of *DNMT3A* knockouts become only apparent after even longer culturing periods. Either way, a moderate growth advantage of clones with specific aberrations in *DNMT3A* might be a functional correlate to malignant transformation.

## Conclusions

Hematopoietic differentiation is hardly affected in iPSCs with homozygous knockout of exon 19 or 23, despite almost entire absence of de novo methylation. This was unexpected given the functional relevance of DNAm during differentiation. It will therefore be important to gain better insight into cell-type specific hypomethylation and the interplay with other epigenetic modifications. Our model system can help to get a better understanding on the relevance of DNMT3A during hematopoietic development and malignant transformation.

## Methods

### iPSC lines, ethics approval, and consent to participate

iPSCs of donor 1 and donor 2 were reprogrammed from MSC preparations with episomal plasmids and characterized, as described before [38]. iPSCs of donor 3 were generated from KIT+ cells from bone marrow aspirates of a mastocytosis patient, with the CytoTune-iPS Sendai Reprogramming Kit (Thermo Fisher Scientific, Waltham, MA, USA). This line was also carefully characterized and revealed a *KRAS* mutation A146T. All samples were taken after informed and written consent and the study was approved by the Ethic Committee of the Use of Human Subjects at the University of Aachen (permit numbers: EK128/09 and EK206/09, respectively). iPSCs were cultured on tissue culture plastic coated either with vitronectin (0.5 mg/cm^2^; Stemcell Technologies, Vancouver, Canada) or Matrigel Matrix (Corning, Corning, NY, USA) in StemMACS iPS-Brew XF (Miltenyi Biotec, Bergisch Gladbach, Germany) with 100 U/mL penicillin and 100 μg/mL streptomycin. Pluripotency was validated by Epi-Pluri-Score (Cygenia GmbH, Aachen, Germany) [23].

### CRISPR/Cas9n knockout of *DNMT3A*

Knockouts of exon 2, 19, and 23 of *DNMT3A* were generated with CRISPR/Cas9n double nicking approach [39]. Two pairs of gRNAs were designed with the help of the CRISPOR tool [40] (Additional file 1: Fig. S1a & Tab. S6). Intron/exon boundaries of exon 2 and exon 19 were targeted in order to achieve altered splicing via exon skipping. Complete excision of exon 23 was performed as this exon did not allow for design of effective gRNAs for exon/intron boundary targeting. gRNA primers were individually ligated into a variant of the pX335 vector (Addgene #42335) additionally containing the reporter protein GFP and a puromycin selection cassette [41, 42]. Transfection of 10^6^ iPSCs with the four gRNA plasmids (2.5 μg each) was performed with the NEON transfection system kit (Thermo Fisher Scientific) and after 1 day transfected cells were selected with 0.4 μg/mL puromycin for 48 h. Single colonies were picked and screened for *DNMT3A* deletions by PCR and Sanger sequencing (primers: Additional file 1: Tab. S7). Knockout was further confirmed with semi-quantitative real-time PCR (primers: Additional file 1: Tab. S8) and Western blots; pluripotency was tested with immunofluorescence and trilineage differentiation assays (qPCR primers for trilineage analysis: Additional file 1: Tab. S9) as described in Additional file 1.

### Differentiation of iPSCs into the mesenchymal lineage

For iMSC generation, iPSC medium was switched to MSC medium, consisting of Dulbecco’s modified Eagle’s medium-low glucose (Thermo Fisher Scientific) supplemented with 10% human platelet lysate, and 5 U/mL heparin (Rotexmedica, Trittau, Germany) for 35 days, as described before [43]. Surface marker expression was measured with flow cytometry (Additional file 1). We have also analyzed three lineage differentiation potential of iMSCs as described before [43], but due to high variation between cell lines and replicas a reliable quantitative comparison was not feasible.

### Hematopoietic progenitor differentiation

To differentiate iPSCs into hematopoietic progenitors, we adjusted the protocol from Liu et al. [44]: EBs were generated from iPSCs by self-detachment from microcontact-printed vitronectin spots generated with PDMS stamps [45]. EBs were carefully resuspended in serum free medium containing 50% IMDM, 50% Ham’s F12, 0.5% BSA, 1% chemically defined lipid concentrate, 2 mM GlutaMAX (all Thermo Fisher Scientific), 400 μM 1-thioglycerol, 50 μg/mL L-ascorbic acid, and 6 μg/mL holo transferrin (all Sigma Aldrich, St. Louis, MO, USA) supplemented with 10 ng/mL FGF-2 (Peprotech, Hamburg, Germany), 10 ng/mL BMP-4 (Miltenyi Biotec), and 10 μM Y-27632 (Abcam, Cambridge, Great Britain). EBs were seeded on 0.1% gelatin coated 6-well plates. From day 2 to 16, EBs were cultured in serum-free medium supplemented with 10 ng/mL FGF 2, 10 ng/mL BMP-4, 50 ng/mL SCF, 10 ng/mL VEGF-A (all Peprotech), and 10 U/mL penicillin/streptomycin (Thermo Fisher Scientific). The non-adherent iHPCs were harvested at day 16 and separated with a 40 μm cell strainer. Immunophenotype was tested with flow cytometry and stem cell potential with colony forming unit assays as described in Additional file 1.

### DNA methylation analysis

Genomic DNA was isolated with the NucleoSpin Tissue kit (Macherey Nagel) and subsequently bisulfite converted and hybridized to the Infinium MethylationEPIC BeadChip (Illumina, San Diego, California, USA) at Life and Brain GmbH (Bonn, Germany). Data was preprocessed with the Bioconductor Illumina Minfi package for R [46, 47] and normalized with ssnoob. CpGs on X and Y chromosomes, SNPs, and cross-reactive sites were excluded, resulting in 741896 CpGs. For comparison with other datasets (iHPC: GSE60811 [19, 48] and GSE119079 [20, 49]; whole blood, granulocytes, B cells, CD4+ T cells, CD8+ T cells, NK cells, monocytes: all GSE35069 [50, 51]; CD34+ cells isolated from human cord blood: GSE40799 [52, 53]; primary MSCs: GSE113527 [54, 55]; AML data obtained from The Cancer Genome Atlas [56]), we further focused on CpGs that were presented by the Illumina Infinium HumanMethylation450 BeadChip and the Infinium MethylationEPIC BeadChip. Principal component analysis and multidimensional scaling plots were prepared in R using the ggplot2 package version 3.3.3 [57]. Heatmaps were generated with the MultiExperiment Viewer (MeV; version 4.9.0). Gene ontology analysis was performed on genes with differentially methylated CpGs in the promoter region (located in TSS1500, TSS200, and 5′UTR) in R with the missMethyl package [58]. Categories comprising more than 1000 genes were not considered and similar categories are only listed once. Venn diagrams were prepared with the InteractiVenn tool [59]. Motif analysis was performed with the Hypergeometric Optimization of Motif EnRichment (HOMER) suite [60] with a 100 bp frame around hypermethylated CpGs. Canyons were defined as 30 neighboring CpG sites within 3 kb and an average β-value below 0.15. Epigenetic age was predicted using Horvath’s skin and blood clock [61].

### Gene expression analysis

For transcriptomic analysis, total RNA was isolated from iHPCs at day 16 of differentiation with the NucleoSpin RNA Plus kit (Macherey Nagel). mRNA was prepared with the QuantSeq 3’ mRNA-Seq Library Prep Kit FWD for Illumina (Lexogen, Vienna, Austria) and sequenced with 10 million 50 bp single-end reads on the HiSeq 2500v4 (Illumina) at Life and Brain GmbH. Raw data underwent quality control with Trim Galore and sequences were aligned to the human genome (hg38) by bowtie2 [62] aligner with default parameters. Differential expression analysis was done by DESeq2 [63] with the adjusted *p*-value threshold of 0.05. Scatter plots were generated with ggplot2 [57] in R.

### Genetic barcoding of iPSC clones with RGB LeGO vectors

Generation of barcoded RGB vectors was performed as described in detail before [37]. LeGO vectors expressing either the fluorophores Venus, Cerulean, or mCherry (pRRL-PPT-CBX3-EFS-Cerulean-P2A-Puro, pRRL-PPT-CBX3-EFS-mCherry-P2A-Puro, pRRL-PPT-CBX3-EFS-Venus-P2A-Puro [64]) were ligated with unique molecular identifiers containing random as well as color-specific nucleotide sequences [65] (RGB-vectors; kindly provided by the Medizinische Hochschule Hannover, Germany). Wildtype, exon 19^−/−^ and exon 23^−/−^ iPSCs were transduced with the Venus, Cerulean, or mCherry libraries, respectively, and used for a competitive hematopoietic differentiation assay as described in detail in Additional file 1.

### MiSeq analysis of barcoded cells

MiSeq was performed as described in detail before [37]. In brief, DNA was isolated with the NucleoSpin Tissue kit (Macherey Nagel), barcodes were amplified, tagged with Illumina sample barcodes, samples were pooled equimolarly, and a 4 nM library was prepared for sequencing with a 50% spike in of PhiX Control DNA and the MiSeq Reagent Kit v2 (all from Illumina). Libraries were sequenced in 250-bp paired-end mode on an Illumina MiSeq Benchtop Sequencer. Raw data was analyzed with Python. Unique molecular identifiers of the RGB-vectors were extracted from FastQ files and grouped according to color-specific sequences resembling the different cell lines. Two mismatches were allowed for grouping of these color-specific identifiers. Finally, directional clustering from UMI-tools [66] was used to specify unique molecular identifiers for each color-group. Area plots were prepared with ggplot2 in R.

### Statistics and reproducibility

All experiments were performed with three wildtype iPSC lines and two knockout iPSC lines for *DNMT3A* exon 2, 19, and 23. Genetic barcoding experiments were performed with the syngeneic iPSC clones of donor 1 (corresponding wildtype, exon 19 knockout, and exon 23 knockout) in three technical replicates. Data is provided as mean with standard deviation (SD). Significance of flow cytometry data was calculated by 2-way ANOVA with Tukey’s post hoc test. One-way ANOVA with Tukey’s post hoc test was used for iHPC counts, CFU colonies, for comparison of methylation data with AML patients, and for flow cytometry data of RGB cells (GraphPad version 9.1.1.). Significantly differentially expressed genes were tested in R with DESeq2 using the default Wald test. Significance of overlapping hypermethylated CpGs within iMSCs and iHPCs of exon 19 and exon 23 knockout clones was calculated in R with the gmp package via hypergeometric distribution.

## Supplementary Information

**Additional file 1: **Combined PDF of Supplemental figures S1 – S8, Supplemental tables S6 – S9 and Supplemental experimental procedures. **Fig. S1**. Generation of CRISPR/Cas9n knockout iPSC clones. **Fig. S2**. Full western blot images of DNMT3A and actin bands. **Fig. S3**. Pluripotency and global DNA methylation in iPSC lines. **Fig. S4**. Gene ontology analysis and overlap of differentially methylated CpGs upon mesenchymal differentiation. **Fig. S5**. Characterization of iPSC-derived hematopoietic progenitor cells. **Fig. S6**. DNA methylation analysis of hematopoietic progenitor cells. **Fig. S7**. Comparison of iHPCs with exon 19 or exon 23 knockout. **Fig. S8**. Gene methylation, age prediction and canyon analysis of iPSCs, iMSCs and iHPCs. **Tab. S6**. gRNAs for CRISPR. **Tab. S7**. Flanking primers for PCR. **Tab. S8**. Exon specific primers for qPCR. **Tab. S9**. qPCR primers for the trilineage assay.

**Additional file 2: Tab. S1**. Differentially methylated CpGs in iPSC WT *versus DNMT3A* knockouts. CpG sites that are either 50% hypo- or hypermethylated in *DNMT3A* exon 2^-/-^, exon 19^-/-^, or exon 23^-/-^ iPSCs compared to wildtype iPSCs with associated genes, gene groups, relation to CpG islands, chromosomes, positions, mean beta-values of the knockout and wildtype cells, and the difference in methylation between knockout and wildtype.

**Additional file 3: Tab. S2**. Differentially methylated CpGs in iPSCs *versus* iMSCs (WT or *DNMT3A* knockouts). CpG sites that are either 50% hypo- or hypermethylated in *DNMT3A* wildtype, exon 2^-/-^, exon 19^-/-^, or exon 23^-/-^ iMSCs compared to their iPSC counterparts with associated genes, gene groups, relation to CpG islands, chromosomes, positions, mean beta-values of the knockout and wildtype cells, and the difference in methylation between knockout and wildtype.

**Additional file 4: Tab. S3**. Differentially methylated CpGs in iPSCs *versus* iHPCs (WT or *DNMT3A* knockouts). CpG sites that are either 50% hypo- or hypermethylated in *DNMT3A* wildtype, exon 19^-/-^, or exon 23^-/-^ iHPCs compared to their iPSC counterparts with associated genes, gene groups, relation to CpG islands, chromosomes, positions, mean beta-values of the knockout and wildtype cells, and the difference in methylation between knockout and wildtype.

**Additional file 5: Tab. S4**. Differentially methylated CpGs in iHPC WT *versus DNMT3A* knockouts. CpG sites that are either 50% hypo- or hypermethylated in *DNMT3A* exon 19^-/-^, or exon 23^-/-^ iHPCs compared to wildtype iHPCs with associated genes, gene groups, relation to CpG islands, chromosomes, positions, mean beta-values of the knockout and wildtype cells, and the difference in methylation between knockout and wildtype.

**Additional file 6: Tab. S5**. RNA-seq data of iHPCs. Genes that are either four-fold downregulated or upregulated (log_2_ fold change <-2 or >2) in *DNMT3A* wildtype *versus* exon 19^-/-^, wildtype *versus* exon 23^-/-^, or exon 19^-/-^ *versus* exon 23^-/-^ with gene name, base mean, log_2_ fold change, *P*-value, adjusted *P*-value, and read counts per million of knockout and control cells.

## Data Availability

All data generated or analyzed during this study are included in this published article, its supplementary information files and publicly available repositories. Individual data values are available upon reasonable request. Data of DNA methylation profiles generated in this study has been deposited at Gene Expression Omnibus (GEO) under the reference ID GSE180402 [67]. RNA-Seq data has been deposited at GEO under the reference ID GSE180403 [68]. Publicly available datasets analyzed in this study are accessible at GEO with the reference ID GSE60811 [19, 48] and GSE119079 [20, 49] for iHPCs; GSE35069 [50, 51] for whole blood, granulocytes, B cells, CD4+ T cells, CD8+ T cells, NK cells, and monocytes; GSE40799 [52, 53] for CD34+ cells isolated from human cord blood; and GSE113527 [54, 55] for primary MSCs. Methylation data of acute myeloid leukemia patients was taken from The Cancer Genome Atlas [56].

## Abbreviations

AML: Acute myeloid leukemia
CFU: Colony forming unit
CHIP: Clonal hematopoiesis of indeterminate potential
CpG site: Cytosine-Guanine dinucleotide
DNAm: DNA methylation
EB: Embryoid body
ESC: Embryonic stem cell
Ex: Exon
gRNA: Guide RNA
HSC: Hematopoietic stem cell
iHPC: Induced pluripotent stem cell-derived hematopoietic progenitor cell
iMSC: Induced pluripotent stem cell-derived mesenchymal stromal cell
iPSC: Induced pluripotent stem cell
MDS: Multidimensional scaling
MSC: Mesenchymal stromal cell
PCA: Principal component analysis
PDMS: Polydimethylsiloxane
RGB: Red green blue
RT-PCR: Real-time polymerase chain reaction
SD: Standard deviation
TCGA: The Cancer Genome Atlas
TSS: Transcription start site
WT: Wildtype
