# Supplementary material for: Hematopoietic differentiation persists in human iPSCs defective in de novo DNA methylation
Source: BMC Biol. 2022 Jun 15;20:141. doi: 10.1186/s12915-022-01343-x (PMC9202186; doi:10.1186/s12915-022-01343-x)
Supplement: Supplementary file 1 — Additional file 1: Combined PDF of Supplemental figures S1 – S8, Supplemental tables S6 – S9 and Supplemental experimental procedures. Fig. S1. Generation of CRISPR/Cas9n knockout iPSC clones. Fig. S2. Full western blot images of DNMT3A and actin bands. Fig. S3. Pluripotency and global DNA methylation in iPSC lines. Fig. S4. Gene ontology analysis and overlap of differentially methylated CpGs upon mesenchymal differentiation. Fig. S5. Characterization of iPSC-derived hematopoietic progenitor cells. Fig. S6. DNA methylation analysis of hematopoietic progenitor cells. Fig. S7. Comparison of iHPCs with exon 19 or exon 23 knockout. Fig. S8. Gene methylation, age prediction and canyon analysis of iPSCs, iMSCs and iHPCs. Tab. S6. gRNAs for CRISPR. Tab. S7. Flanking primers for PCR. Tab. S8. Exon specific primers for qPCR. Tab. S9. qPCR primers for the trilineage assay. [file 12915_2022_1343_MOESM1_ESM.pdf]

Additional information

Hematopoietic differentiation persists in human iPSCs defective in *de novo* DNA methylation

Olivia Cypris, Julia Franzen, Joana Frobel, Philipp Glück, Chao-Chung Kuo, Stephani Schmitz, Selina Nüchtern, Martin Zenke, and Wolfgang Wagner

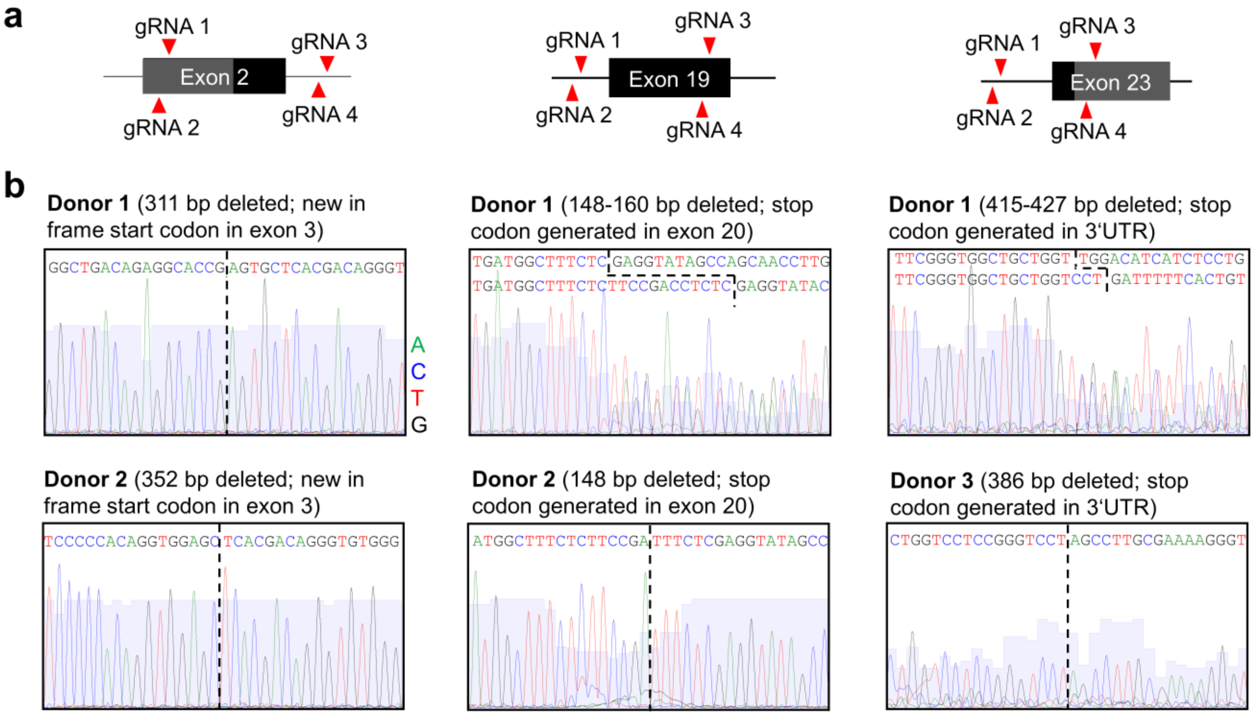

Fig. S1: Generation of CRISPR/Cas9n knockout iPSC clones.

(a) Schematic representation of gRNA design for exon 2 knockout, exon 19 knockout, and exon 23 knockout, respectively. The boundary between the grey and black box of exon 2 is the transcription start site. Red arrows depict cutting sites of guide RNAs (gRNAs). **(b)** Sanger sequencing results are depicted for the two clones for each exon, which were subsequently used in this study. Dotted lines = CRISPR cutting site. In donor 1 exon 19 and exon 23, each allele was cut at different sites as indicated.

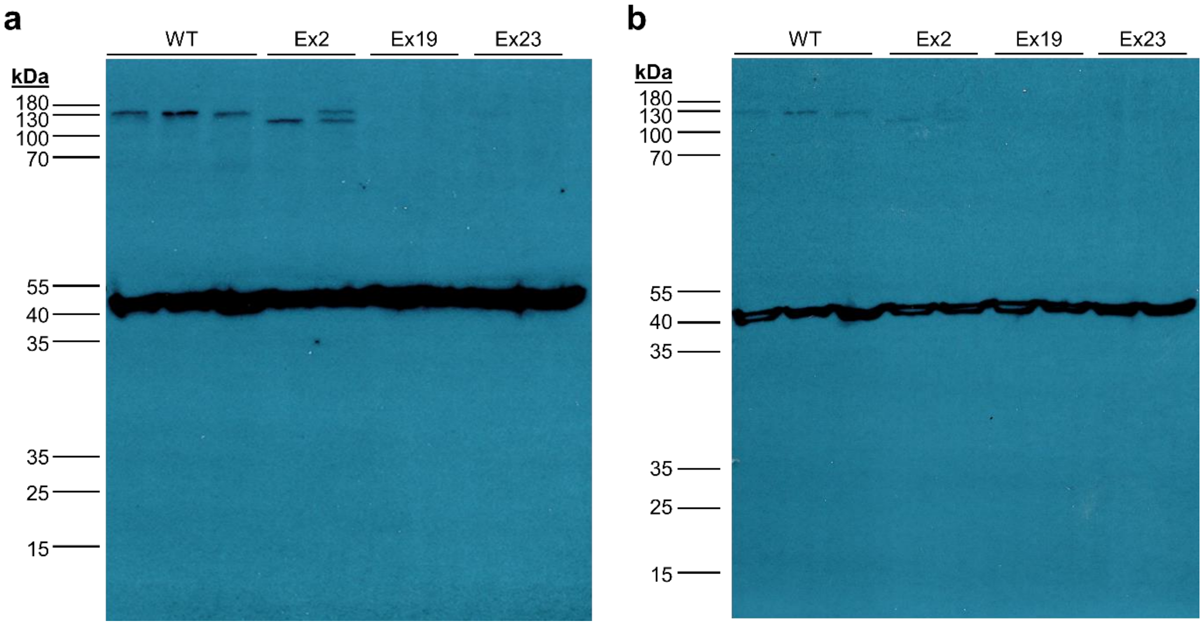

Fig. S2: Full western blot images of DNMT3A and actin bands.

(a) Western blot analysis of DNMT3A upon knockout of exon 2, exon 19, or exon 23. Bands around 130 kDa correspond to height of transcript 1 / 3 while bands around 45 kDa correspond to actin. CRISPR knockout of exon 2 results in a truncated protein that is predicted to be 8.5 kDa smaller than the wildtype protein with a total size of around 121.5 kDa. X-ray film was exposed for two minutes. WT = wildtype. **(b)** Similar to (a) with an exposure time of 30 seconds.

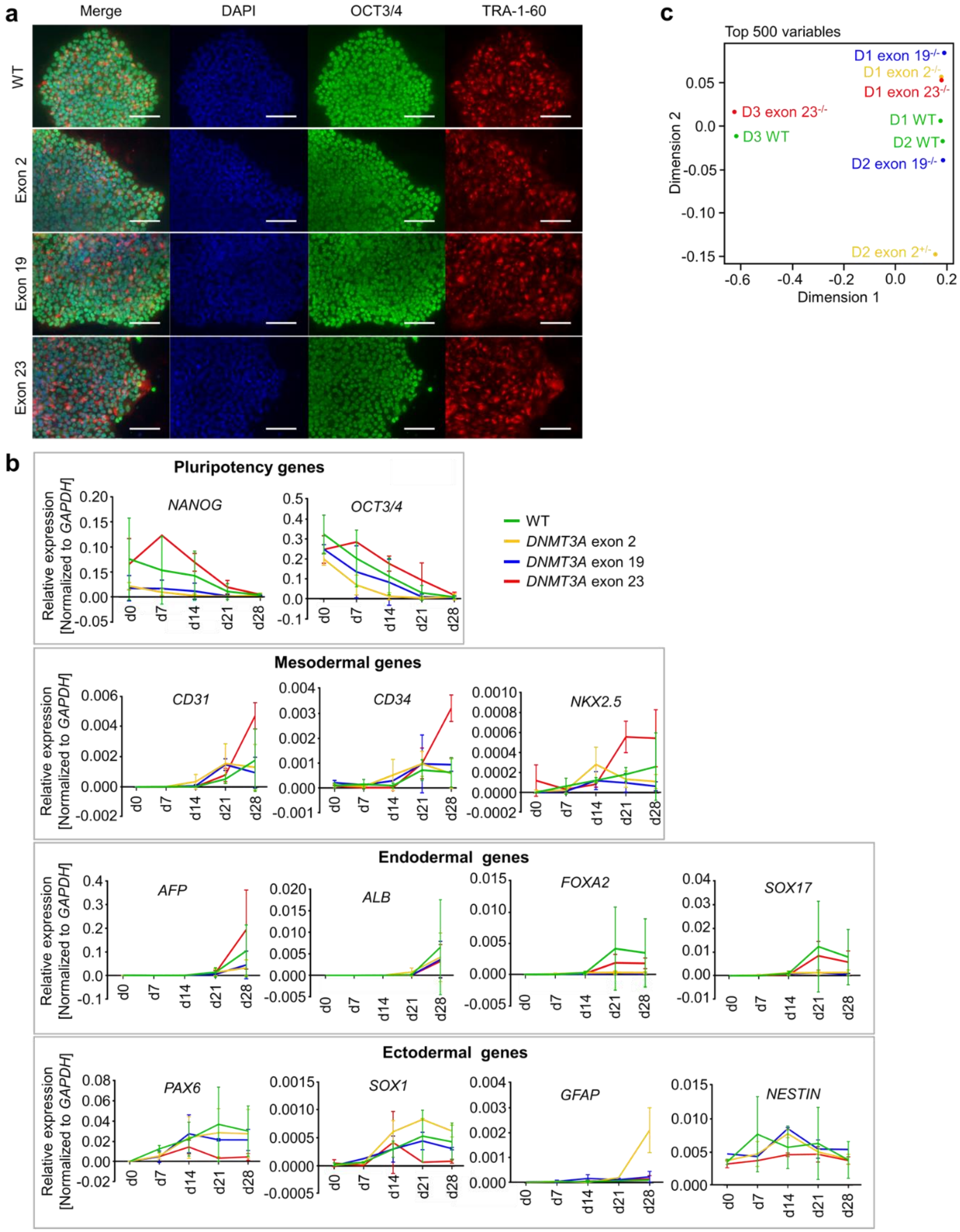

Fig. S3: Pluripotency and global DNA methylation in iPSC lines.

(a) Immunofluorescence staining of nuclei with DAPI (blue) and the pluripotency markers OCT3/4 (green) and TRA-1-60 (red) of representative clones of wildtype (WT) and knockout lines. **(b)** Relative expression of pluripotent, mesodermal, endodermal and ectodermal markers after trilineage differentiation of all iPSC lines as measured by quantitative real-time PCR. Expression values were normalized to the housekeeping gene *GAPDH*. n = 3 for WT, n = 2 for each knockout with duplicates for each measurement. Data is presented as mean ± SD. **(c)** Multidimensional scaling plot of all iPSC lines with the top 500 most variable CpG sites (D = donor).

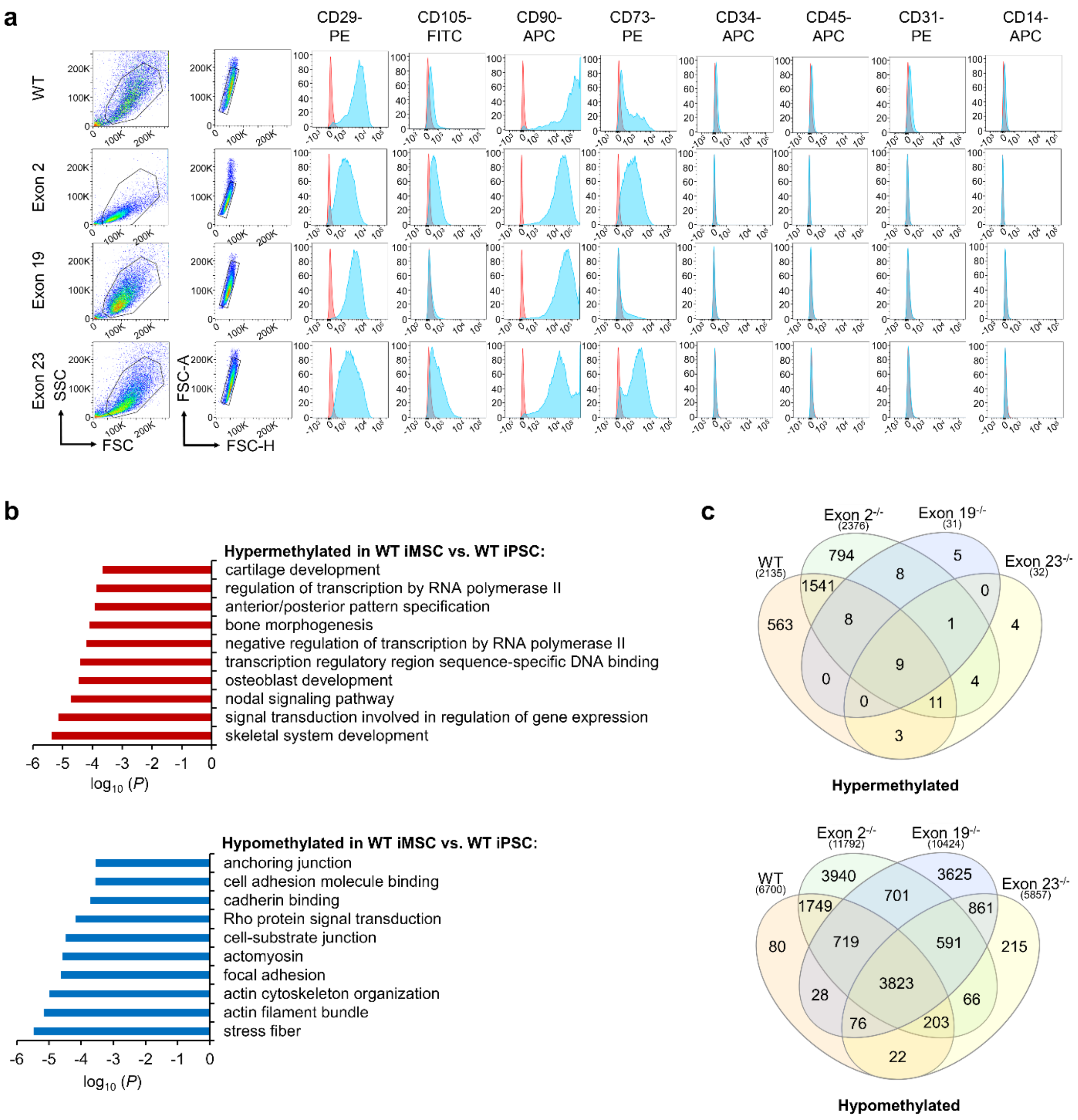

Fig. S4: Gene ontology analysis and overlap of differentially methylated CpGs upon mesenchymal differentiation.

(a) Flow cytometry analysis of iMSCs after 35 days of differentiation. WT = wildtype, FSC = forward scatter, SSC = side scatter. **(b)** Gene ontology analysis of genes with >50% differentially methylated CpG sites in the promoter region. **(c)** Venn diagram showing the overlap of 50% hyper- (left) and hypomethylated (right) CpG sites during differentiation of iPSCs to iMSCs, when comparing wildtype (WT) and *DNMT3A* knockout clones (means of two biological replica per comparison for exon $19^{-/-}$ and exon $23^{-/-}$).

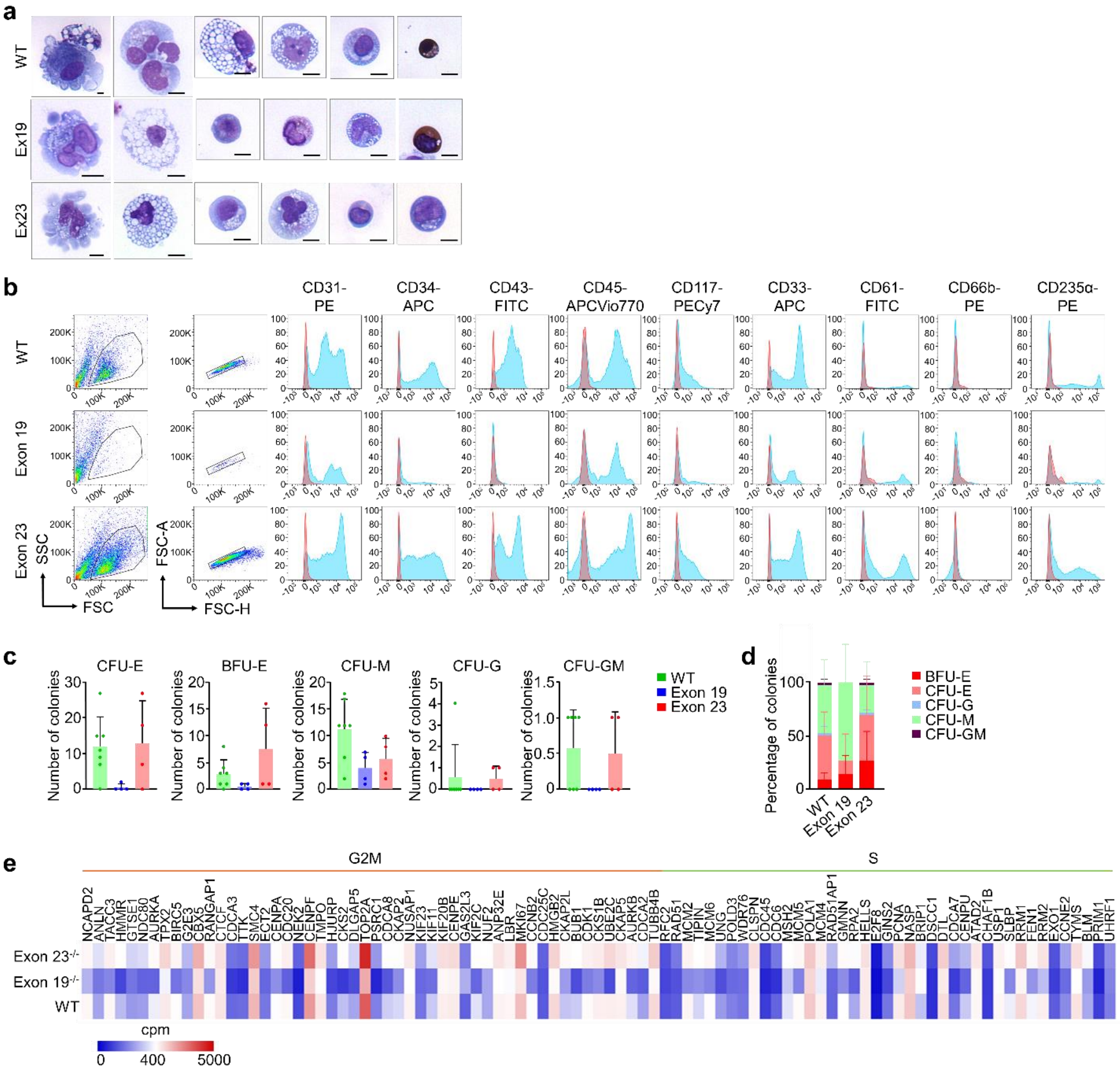

Fig. S5: Characterization of iPSC-derived hematopoietic progenitor cells.

(a) Cytospins of iHPCs on day 16 of hematopoietic differentiation point toward hematopoietic morphologies in all wildtype and *DNMT3A* knockout clones. Scale bar = 10 µm. **(b)** Flow cytometry analysis of iHPCs after 16 days of differentiation. WT = wildtype, FSC = forward scatter, SSC = side scatter. **(c)** Total numbers of colony forming units (CFUs) for each colony type. CFU-E = CFU erythrocyte CFU; BFU-E = burst forming unit erythrocyte; CFU-M = CFU macrophage; CFU-G = CFU granulocyte; CFU-GM = CFU granulocyte, macrophage; CFU-GEMM = granulocyte, erythrocyte, macrophage, megakaryocyte. **(d)** Proportion of CFU colonies in WT, exon 19 knockout and exon 23 knockout clones. Statistics for (c) and (d) were calculated with 1-way ANOVA and Tukey's PostHoc test. n = 7 for WT, n = 4 for each knockout with duplicates for each measurement. Data is presented as mean ± SD. **(e)** Heatmap showing the gene expression of cell cycle related genes (G2M phase and S phase) in WT, exon 19 and exon 23 knockout iHPCs. The mean of three replica for WT and two replica for each knockout is provided. In tendency the proliferation associated genes are less expressed in exon $19^{-/-}$ cells, but none of the genes showed significant differences. cpm = counts per million in RNA-seq data.

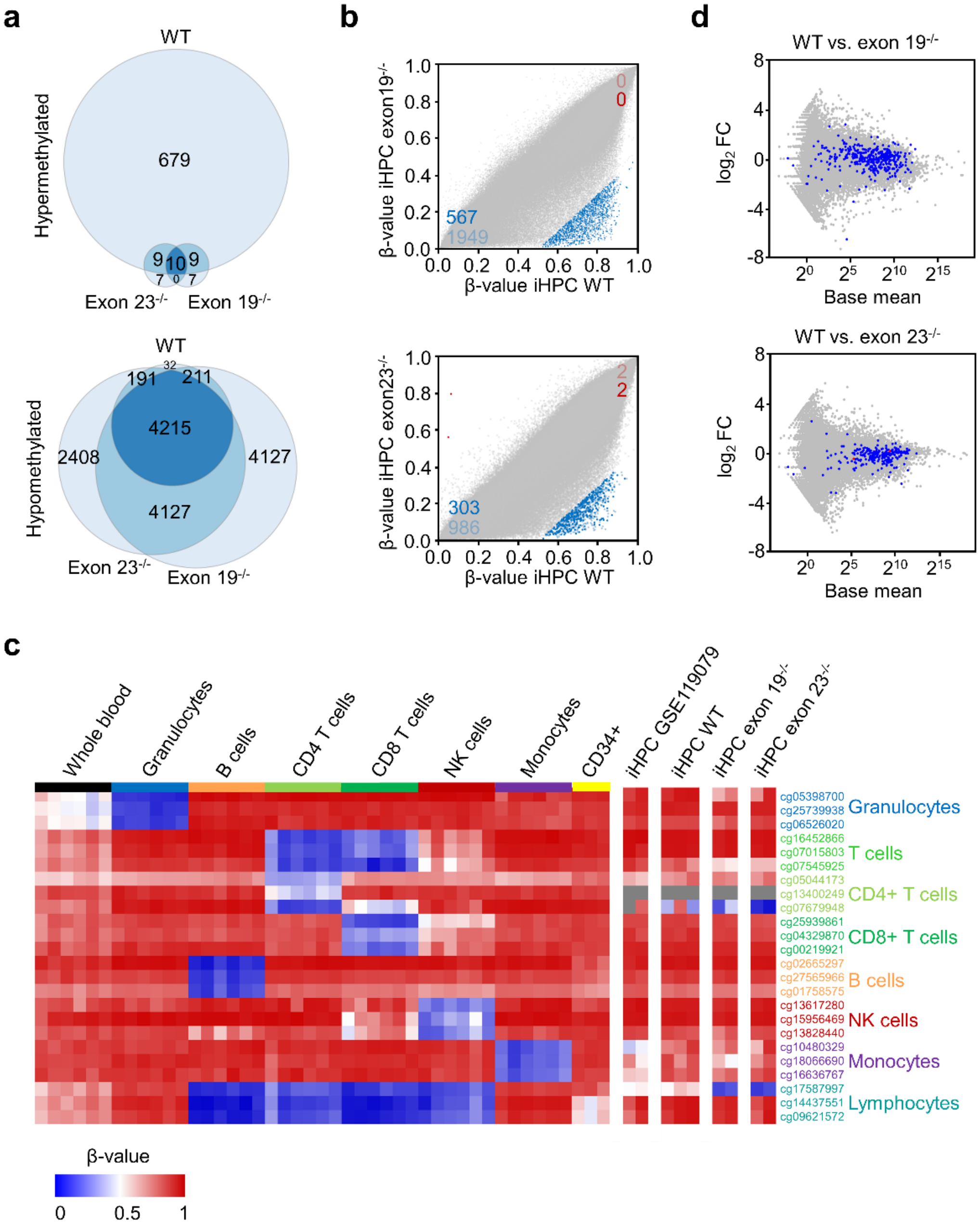

Fig. S6: DNA methylation analysis of hematopoietic progenitor cells.

(a) Venn diagram showing the overlap of CpG sites that become at least 50% hyper- or hypomethylated during differentiation of iPSCs into iHPCs (mean of two replica for each comparison). WT = wildtype. **(b)** Scatter plots showing β-values for all CpG sites measured in iHPC wildtypes and exon 19 or exon 23 knockouts in grey, 50% hypo- (blue) and 50% hypermethylated (red) CpG sites. The darker blue and red dots and numbers represent CpG sites in the promoter region. **(c)** Heatmap of DNA methylation levels at CpGs that are specifically hypomethylated in specific hematopoietic cell types. The profiles of whole blood, granulocytes, B cells, CD4+ T cells, CD8+ T cells, NK cells, monocytes were taken from GSE35069 (1); CD34+ cells isolated from human cord blood from GSE40799 (2); and DNAm profiles of iHPC from GSE119079 (3) and our current study. Gray areas indicate missing data. **(d)** Differentially promoter-methylated CpG associated genes in iHPC wildtypes compared to exon $19^{-/-}$ or exon $23^{-/-}$ are plotted in blue (50% hypomethylated) or red (50% hypermethylated) in the graphs from Fig. 3h. See also Table S8.

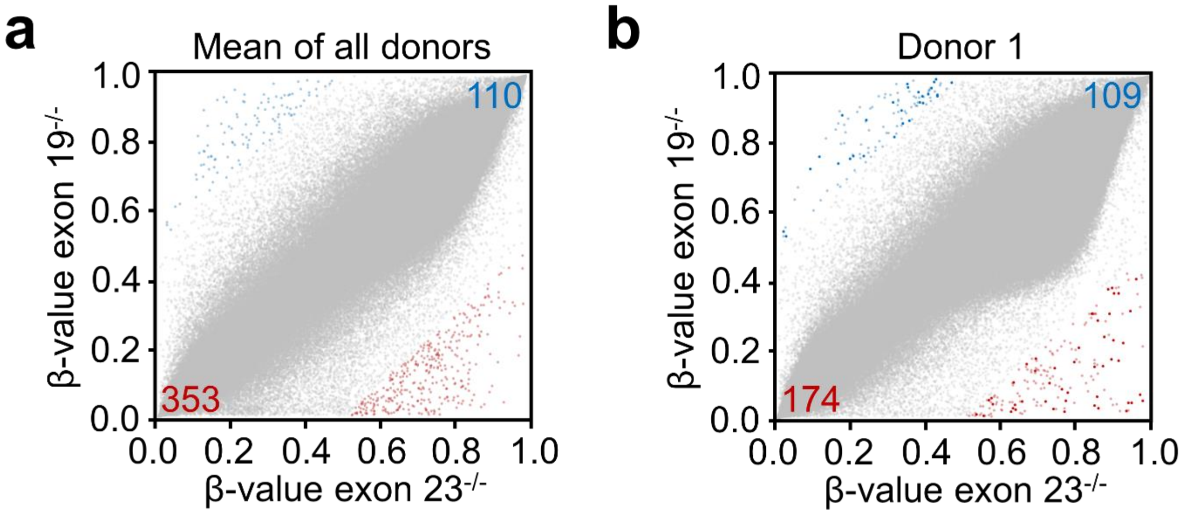

Fig. S7: Comparison of iHPCs with exon 19 or exon 23 knockout.

(a) Scatter plot showing mean β-values for all CpG sites measured in iHPC exon 19 and exon 23 knockout cells of all donors in grey with 50% hypo- and 50% hypermethylated CpG sites indicated in blue or red, respectively. **(b)** Same as (a) but showing values for the comparison of donor 1 clones only.

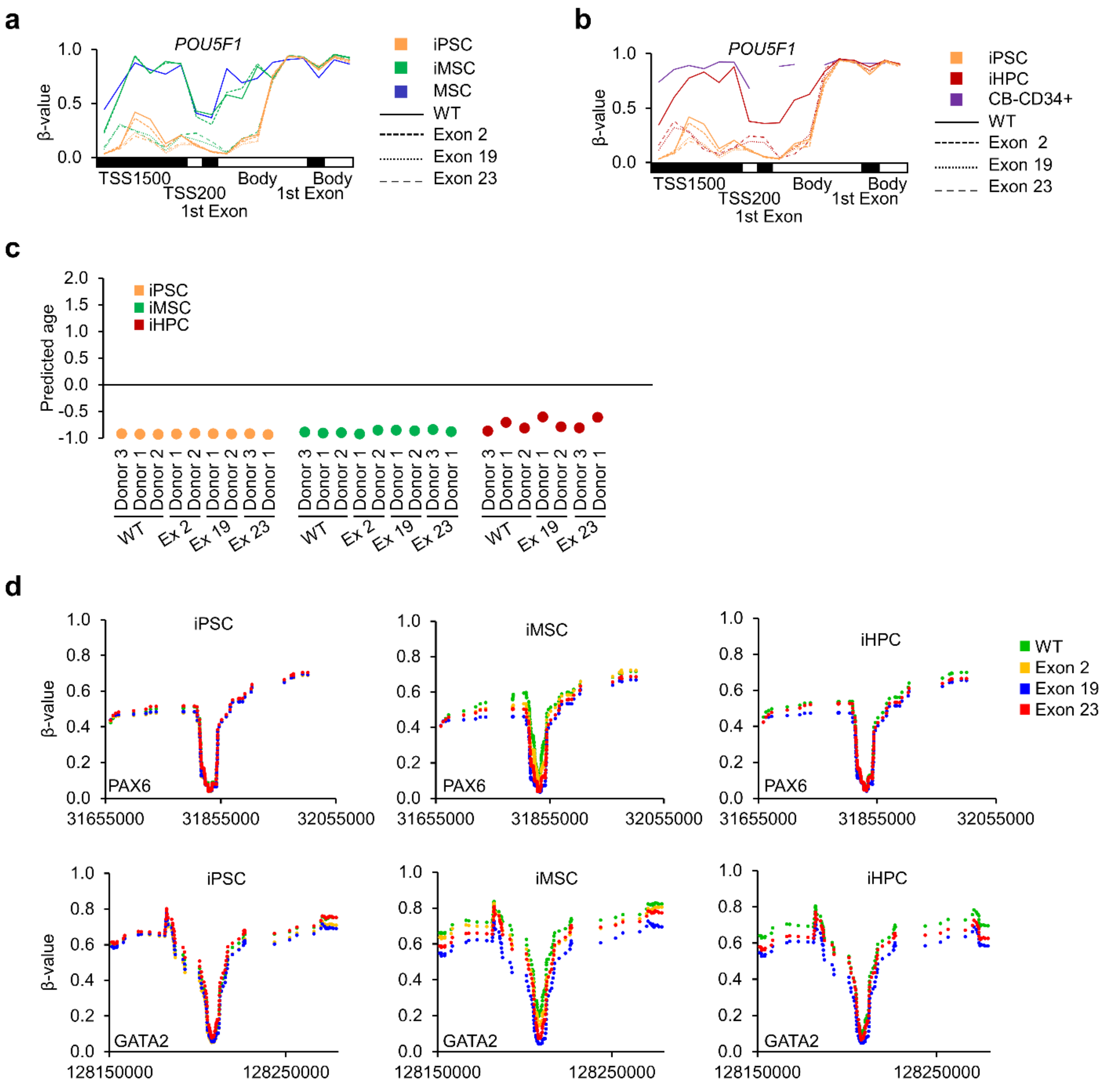

Fig. S8: Gene methylation, age prediction and canyon analysis of iPSCs, iMSCs and iHPCs.

(a) DNAm of CpGs within the gene of *POU5F1* in iPSCs, iMSCs, and primary MSCs (GSE113527) (4). n = 2 for primary MSC and each knockout, n = 3 for wildtype (WT). Data is presented as mean**.** **(b)** DNAm of CpGs within the gene *POU5F1* in iPSCs, iHPCs, and primary cord-blood (CB) derived CD34+ cells (GSE40799) (2). n = 1 for CB-CD34+, n = 3 for WT, n = 2 for each knockout. Data is presented as mean. **(c)** Epigenetic age was predicted with Horvath‘s clock (Horvath et al., 2018) for iPSCs, iMSCs, and iHPCs. **(d)** Canyons were analyzed in the genes *PAX6*, and *GATA2* for iPSCs, iMSCs, and iHPCs. The two exemplary genes were selected for comparison to Jeong et al., 2018. n = 3 for WT, n = 2 for each knockout. Data is presented as mean**.**

Additional tables

Table S1: Differentially methylated CpGs in iPSC WT *versus DNMT3A* knockouts.

This table is provided as Additional file 2.xlsx

Table S2: Differentially methylated CpGs in iPSCs *versus* iMSCs (WT or *DNMT3A* knockouts).

This table is provided as Additional file 3.xlsx

Table S3: Differentially methylated CpGs in iPSCs *versus* iHPCs (WT or *DNMT3A* knockouts).

This table is provided as Additional file 4.xlsx

Table S4: Differentially methylated CpGs in iHPC WT *versus DNMT3A* knockouts.

This table is provided as Additional file 5.xlsx

Table S5: RNA-seq data of iHPCs.

This table is provided as Additional file 6.xlsx

Table S6: gRNAs for CRISPR.

Primer	Sequence (5' – 3')
Exon 2_1a FW	CACCGACAGAGGCACCGTTCACCAG
Exon 2_1a RV	AAACCTGGTGAACGGTGCCTCTGTC
Exon 2_1b FW	CACCGCCTGTGGGTGGGGGCTTCGA
Exon 2_1b RV	AAACTCGAAGCCCCCACCCACAGGC
Exon 2_2a FW	CACCGATCACTCAGTGCTCACGACA
Exon 2_2a RV	AAACTGTCGTGAGCACTGAGTGATC
Exon 2_2b FW	CACCGGCGGTCATGCACTCAGTATG
Exon 2_2b RV	AAACCATACTGAGTGCATGACCGCC
Exon 19_1a FW	CACCGTTTCTCTTCCGACCTCTCAG
Exon 19_1a RV	AAACCTGAGAGGTCGGAAGAGAAAC
Exon 19_1b FW	CACCGCAGCTGGGGCTGTCTGCAT
Exon 19_1b RV	AAACATGCAGACAGCCCCAGCTGC
Exon 19_2a FW	CACCGGGACATCTCGCGATTTCTCG
Exon 19_2a RV	AAACCGAGAAATCGCGAGATGTCCC
Exon 19_2b FW	CACCGCCTCTTGTCACTAACGCCCA
Exon 19_2b RV	AAACTGGGCGTTAGTGACAAGAGGC
Exon 23_1a FW	CACCGTAGACGGCTTCCGGGCAGCC
Exon 23_1a RV	AAACGGCTGCCCGGAAGCCGTCTAC
Exon 23_1b FW	CACCGAACCACACAGCAGGACCCGG
Exon 23_1b RV	AAACCCGGGTCCTGCTGTGTGGTTC
Exon 23_2a FW	CACCGCTTTGCCTTGCGAAAAGGGT
Exon 23_2a RV	AAACACCCTTTTCGCAAGGCAAAGC
Exon 23_2b FW	CACCGCTCCGGTATTTCCGCCTCTG
Exon 23_2b RV	AAACCAGAGGCGGAAATACCGGAGC

Table S7: Flanking primers for PCR.

Primer	Sequence (5' – 3')
Exon 2 FW	GGTTTCTCTGCTCTCTGGGGTG
Exon 2 RV	CTGGAACCAAATGCCTGCTCC
Exon 19 FW	GCAATGACCTCTCCATCGTCA
Exon 19 RV	CCCTGCTTCCTCCCTTTCTATC
Exon 23 FW	GAAACCAGCACAGTGCCTGG
Exon 23 RV	GAAGGGGGAGGAAGGGAA

Table S8: Exon specific primers for qPCR.

Primer	Sequence (5’ – 3’)
Exon 2-3 FW	CGGGGACACCAGCAGCTC
Exon 2-3 RV	GCTTGCGCTTCCTCCCAG
Exon 16-17 FW	GGTGCTGTCTCTCTTTGATGGA
Exon 16-17 RV	CCGACGTACATGATCTTCCCC
Exon 17/18-19 FW	GAAGCATATCCAGGAGTGGGG
Exon 17/18-19 RV	CAGGAGGCGGTAGAACTCAAA
Exon 22-23 FW	AGGGCAAAGACCAGCATTTTC
Exon 22-23 RV	CCAAGCGGCTCATGTTGGAG
GAPDH FW	TTCGTCATGGGTGTGAACCA
GAPDH RV	CTGTGGTCATGAGTCCTTCCA

Table S9: qPCR primers for the trilineage assay.

Primer	Sequence (5’ – 3’)
OCT3/4 FW	GGGGGTTCTATTTGGGAAGGTA
OCT3/4 RV	ACCCACTTCTGCAGCAAGGG
NANOG FW	CAGAAGGCCTCAGCACCTAC
NANOG RV	ATTGTTCCAGGTCTGGTTGC
CD34 FW	TGGACCGCGCTTTGCT
CD34 RV	CCCTGGGTAGGTAACTCTGGG
NKX2.5 FW	ACCTCAACAGCTCCCTGACTCT
NKX2.5 RV	ATAATCGCCGCCACAAACTCTCC
MYH6 FW	AAGCTCAAGAACGCCTAC
MYH6 RV	CATTCTTTCCTCCTTCTCC
AFP FW	GCCAAGCTCAGGGTGTAG
AFP RV	CAATGACAGCCTCAAGTTGT
ALB FW	GGTGTGTTTCGTCGAGATG
ALB RV	ACTGAGCAAAGGCAATCAAC
CD31 FW	GAGTCCTGCTGACCCTTCTG
CD31 RV	ATTTTGCACCGTCCAGTCC
FOXA2 FW	GCATTCCCAATCTTGACACGGTGA
FOXA2 RV	GCCCTTGCAGGCAGAATACACATT
SOX17 FW	AGGAAATCCTCAGACTCCTGGGTT
SOX17 RV	CCCAAACTGTTCAAGTGGCAGACA
GFAP FW	AGGAGGAGGTTCGGGAACTC
GFAP RV	CGCCATTGCCTCATACTGC
NESTIN FW	CCTCAAGATCTCCCTCAGCC
NESTIN RV	CCAGCTTGGGGTCCTGAAAG
PAX6 FW	TCGAAGGGCCAAATGGAGAAGAGAAG
PAX6 RV	GGTGGGTTGTGGAATTGGTTGGTAGA
SOX1 FW	CCTGTGTGTACCCTGGAGTTTCTGT
SOX1 RV	TGCACGAAGCACCTGCAATAAGATG
GAPDH FW	GAAGGTGAAGGTCGGAGTC
GAPDH RV	GAAGATGGTGATGGGATTTC

Additional methods

Western blot

To validate *DNMT3A* knockouts, cells were lysed with RIPA buffer and 60 µg protein lysate was loaded onto 12% Mini-PROTEAN TGX Precast Protein Gels (BioRad, Hercules, California, USA). Membranes were blocked with 4% skim milk (Sigma-Aldrich) in TBST for 1 h at room temperature and then incubated with the primary DNMT3A antibody (1:1,000; #2160, Cell Signaling Technology, Danvers, MA, USA) and with the β-actin antibody (1:10,000; clone AC-74, Sigma Aldrich, St. Louis, MO, USA) at 4°C overnight. As secondary antibodies Peroxidase AffiniPure Goat Anti-Rabbit IgG (H+L) (1:5,000; Jackson Immuno Research, West Grove, Pennsylvania, USA) and Goat IgG anti-mouse IgG (H+L)-HRPO (1:10,000; Dianova, Hamburg, Germany) were used and incubated for 1 h at room temperature. Detection was performed with the Super Signal West Dura Extended Duration Substrate (Thermo Fisher Scientific) and X-ray films (CL-XPosure, Thermo Fisher Scientific) with the Optimax 2010 x-ray film processor (Protec, Oberstenfeld, Germany).

Real-time PCR

For semiquantitative real-time PCR (qRT-PCR), RNA was isolated with the NucleoSpin RNA Plus kit (Macherey Nagel). 500 ng RNA was reverse transcribed using the High-Capacity cDNA Reverse Transcription Kit (Thermo Fisher Scientific) and amplified with the StepOnePlus Real-Time PCR system using the Power SYBR Green PCR Master Mix (both Thermo Fisher Scientific) with specific primers (Tab. S8,S9). Expression levels were normalized to *GAPDH*.

Immunofluorescence

For immunofluorescence staining, iPSCs were seeded onto glass slides coated with vitronectin, cultivated until reaching a sufficient colony size, fixed with 4% paraformaldehyde, blocked with normal goat serum for 30 minutes and stained with TRA-1-60 antibody (clone TRA-1-60, Merck Millipore, Burlington, MA, USA) or OCT4 antibody (clone sc-9081, Santa Cruz Biotechnology, Dallas, TX, USA) over night at 4°C. As secondary antibodies goat anti-mouse IgM Alexa Fluor 594 and goat anti-rabbit IgG FITC (both Thermo Fisher Scientific) were used respectively. Nuclei were counterstained with DAPI for 15 minutes. Microscopic pictures were taken with the fluorescence microscope Axioplan 2 (Carl Zeiss, Oberkochen, Germany).

Differentiation in embryoid bodies

For spontaneous trilineage differentiation, iPSCs were seeded in three different densities (1,500; 3,000 and 5,000 cells per well) into U-bottom 96-well plates (TPP, Trasadingen, Switzerland). Plates were centrifuged for 5 minutes at 350 x g and then incubated at 37° C and 5% CO_2 in iPS Brew XF with ROCK-inhibitor and 0.4% polyvinylalcohol (PVA). After 24 h, medium was changed to EB-medium consisting of 77% KO-DMEM, 5% KO serum replacement, 100 U/mL penicillin, 100 µg/mL streptomycin, 1% non-essential amino acids and 0.5% β-mercaptoethanol (all Thermo Fisher Scientific) with daily medium changes. After 7 days EBs were transferred to 0.1% gelatin coated 6-well plates with in total around 30 EBs per well and cultured until day 28.

Immunophenotypic analysis

Flow cytometric analysis was performed on a FACS Canto II (BD Biosciences, Franklin Lakes, New Jersey, USA) and analyzed with FlowJo software Version 10.4.2 (FlowJo LLC, Ashland, Oregon, USA). The following antibodies were used for iMSC analysis: CD14-APC (clone M5E2), CD29-PE (clone MAR4), CD31-PE (clone WM59), CD34-APC (clone 581), CD45-APC (clone HI30), CD73-PE (clone AD2), CD90-APC (clone 5E10) (all BD Biosciences), and CD105-FITC (clone MEM-226; ImmunoTools, Friesoythe, Germany). For analysis of iHPCs we used CD3-APC (clone HIT3a), CD31-PE (clone WM59), CD34-APC (clone 581), CD43-FITC (clone 1G10), CD66b-PE (clone G10F5) (all BD Biosciences, Franklin Lakes, USA), CD11c-PE-Cy7 (clone 3.9), HLA-DR-FITC (1:50, clone LN3), CD235a-PE (1:1,000; clone HIR2/GA-R2), cKIT-PE-Cy7 (clone 104D2), (all Thermo Fisher Scientific), CD45-APC-Vio770 (clone 5B1), CD33-APC (clone AC104.3E3) (all Miltenyi Biotec), and CD61-FITC (clone VI-PL2, Biolegend, San Diego, CA, USA). All antibodies were used in a dilution of 1:100 if not stated otherwise.

Colony forming unit assay

After 16 days of differentiation 5,000 iHPCs were seeded in 500 µL of methylcellulose based medium (HSC-CFU lite with EPO; Miltenyi Biotec) in 24-well plates. Colonies were scored after two weeks of culture.

Cytospin

Cellular morphology of iHPCs was analyzed by cytospins with 1% neutral benzidine (Sigma Aldrich) and Diff Quik staining (Medion Grifols Diagnostics, Düdingen, Switzerland). Cytospins were analyzed with a Leica DMRX microscope (Leica Microsystems, Wetzlar, Germany).

Epi-Pluri-Score analysis

The Epi-Pluri-Score analysis allows classification into pluripotent and non-pluripotent cells by targeted analysis of DNAm at two CpG sites that are either associated with the gene ankyrin repeat domain-containing protein 46 (*ANKRD46*) or chromosome 14 open reading frame 115 (*C14orf115*) (5). DNAm was determined with the Illumina EPIC methylation array data. Additional values at the pluripotency master regulator POU Class 5 Homeobox 1 (*POU5F1*) further helps to distinguish between pluripotent and non-pluripotent cells by demarcating early differentiation events (5).

Competitive hematopoietic differentiation using RGB LeGO vectors

Wildtype, exon $19^{-/-}$ and exon $23^{-/-}$ iPSCs were transduced with Venus, Cerulean, or mCherry RGB libraries, respectively. To this end, iPSCs were grown until a confluency of 30-40%. 200 µL of virus suspension supplemented with 8 µg/mL polybrene (Sigma-Aldrich, St. Louis, MO, USA) was added to the culture medium and cells were centrifuged in 6-well plates at 480 rcf for 2 h and incubated for another hour at 37°C and 5% CO_2. Subsequently, virus suspension was replaced with fresh iPS-Brew XF (Miltenyi Biotec). After 24 h, transduction was repeated by incubation of cells with the virus/polybrene mix for 3 h at 37°C and 5% CO_2. Puromycin selection was started 24 h post-transduction for 2-3 days with increasing puromycin concentrations from 1.0 µg/mL to 1.5 µg/mL. After a short expansion phase the barcoded wildtype, exon $19^{-/-}$ and exon $23^{-/-}$ iPSCs (all donor 1 with same genetic background) were mixed at equal cell numbers for a competitive hematopoietic differentiation assay for 15 days. Thereafter, iHPCs were expanded for additional 28 days in StemSpan SFEM (Stemcell Technologies) supplemented with 10 ng/mL fibroblast growth factor 1 (FGF-1), 10 ng/mL SCF, 10 ng/mL TPO (all purchased from Peprotech), 100 U/mL penicillin and 100 µg/mL streptomycin. Flow cytometric analysis of RGB cells was performed with a Cytek Aurora (Cytek Biosciences, Fremon, CA, USA) and analyzed with the FlowJo software Version 10.4.2. Fluorescence images of RGB cells were acquired with a Zeiss Axiovert 200 (Carl Zeiss, Oberkochen, Germany).

Additional references

1. Reinius LE, Acevedo N, Joerink M, Pershagen G, Dahlen SE, Greco D, et al. Differential DNA methylation in purified human blood cells: implications for cell lineage and studies on disease susceptibility. PloS One. 2012;7(7):e41361; doi:10.1371/journal.pone.0041361.

2. Weidner CI, Walenda T, Lin Q, Wolfler MM, Denecke B, Costa IG, et al. Hematopoietic stem and progenitor cells acquire distinct DNA-hypermethylation during in vitro culture. Sci Rep. 2013;3:3372; doi:10.1038/srep03372.

3. Cypris O, Frobel J, Rai S, Franzen J, Sontag S, Goetzke R, et al. Tracking of epigenetic changes during hematopoietic differentiation of induced pluripotent stem cells. Clin Epigenetics. 2019;11(1):19; doi:10.1186/s13148-019-0617-1.

4. De Witte SFH, Peters FS, Merino A, Korevaar SS, Van Meurs JBJ, O'Flynn L, et al. Epigenetic changes in umbilical cord mesenchymal stromal cells upon stimulation and culture expansion. Cytotherapy. 2018;20(7):919-29; doi:10.1016/j.jcyt.2018.05.005.

5. Lenz M, Goetzke R, Schenk A, Schubert C, Veeck J, Hemeda H, et al. Epigenetic biomarker to support classification into pluripotent and non-pluripotent cells. Sci Rep. 2015;5:8973; doi:10.1038/srep08973.
